# In Silico Druggability Assessment of *Escherichia coli* FtsQ Reveals Tractable PPI Interfaces in the Divisome

**DOI:** 10.3390/antibiotics15050430

**Published:** 2026-04-24

**Authors:** Rok Frlan

**Affiliations:** The Department of Pharmaceutical Chemistry, Faculty of Pharmacy, University of Ljubljana, 1000 Ljubljana, Slovenia; rok.frlan@ffa.uni-lj.si; Tel.: +386-1-4769-674

**Keywords:** antimicrobial resistance, Gram-negative bacteria, FtsQ, bacterial cell division, divisome, protein–protein interactions, druggability assessment, structure-based drug design, in silico analysis, pocket mapping, POTRA domain, antibacterial targets

## Abstract

**Background/Objectives**: Due to the widespread problem of antimicrobial resistance (AMR), there is an urgent need to identify new antibacterial targets that act through mechanisms distinct from those of existing antibiotics. One of these targets is the essential cell division protein FtsQ, which is a central hub of the Gram-negative divisome, but the druggability of its extensive protein–protein interaction (PPI) interfaces remains poorly defined. Here, we present a comprehensive structure-based in silico characterization of *Escherichia coli* FtsQ aimed at identifying and prioritizing druggable regions for PPI modulation. **Methods**: We analyzed *E. coli* FtsQ in both apo and complexed states (FtsQB, FtsQL, and FtsQBL) using a combination of pocket-mapping tools (FTMap and SiteMap), evolutionary conservation analysis (ConSurf), and structure property assessment (BLAST, ProBiS) to map and evaluate potential binding pockets of FtsQ protein. **Results**: Eight potential binding sites were predicted across the β and POTRA domains of FtsQ. One previously unreported site within the POTRA domain was prioritized as a candidate site, characterized by favorable druggability scores, strong evolutionary conservation, and a putative role in the FtsQ–FtsW/FtsN/FtsI interaction network. In contrast, two highly conserved sites at the FtsQ–FtsB/FtsL interaction interface were structurally flat, indicating limited suitability for classical small-molecule binding and greater compatibility with alternative modalities such as macrocycles or peptidomimetics. **Conclusions**: Although FtsQ lacks deep canonical binding pockets, this study proposes several conserved and potentially tractable regions as candidate sites, supporting its potential as a non-classical but promising antibacterial target for disrupting bacterial cytokinesis.

## 1. Introduction

Antimicrobial resistance (AMR), driven by antibiotic overuse and misuse, is a major global health crisis recognized by the WHO, UN, European Commission, and forums like the G20 and G8 [1,2]. This “silent pandemic” severely reduces the effectiveness of existing antibiotics, causing higher morbidity, mortality, longer hospital stays, and rising costs. Without effective intervention, WHO predicts that by 2050, AMR could claim over 10 million lives annually, which is more than cancer, and could result in cumulative economic losses exceeding USD 100 trillion [3]. Meanwhile, the antibiotic pipeline is alarmingly insufficient to combat AMR due to high research and development costs, inadequate investment, and poor financial returns. The majority of recently approved antibiotics are mostly derivatives of older classes, already hampered by the AMR [4,5]. New antibacterial agents with unique mechanisms of action and/or acting on underexplored targets are thus urgently needed to address this growing crisis.

Among these, targeting bacterial cell division stands out as a highly promising therapeutic strategy. This tightly regulated, energy-intensive process is essential for accurate genome partitioning and the physical separation of daughter cells, making it critical for bacterial survival and proliferation [6]. This process is mediated by the divisome, a dynamic and hierarchical assembly of membrane-associated proteins that assemble at midcell and orchestrate the septum formation, membrane remodeling, and peptidoglycan (PG) synthesis, ensuring structural integrity and genetic fidelity during cell division. This complex not only facilitates the physical segregation of the cells but also performs a pivotal function in the coordination of various cellular processes, such as DNA replication and cell wall biosynthesis, thereby guaranteeing that each daughter cell acquires the requisite components necessary for viability [7,8]. Although the mechanism of cell division exhibits slight variations between Gram-positive (G+) and Gram-negative (G−) bacteria, the divisome is a conserved structure characteristic of both types of bacterial species [9].

**Figure 1:**
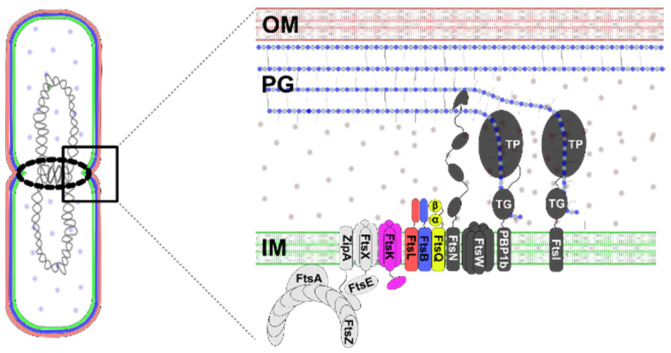
Scheme of the divisome complex showing key subunits. Early proteins (light gray) assemble at the midcell. FtsA and ZipA anchor the Z ring, supporting peptidoglycan synthesis. FtsK (magenta) aids chromosome segregation and recruits the FtsQBL complex (red, blue, yellow), which coordinates division by linking early proteins with late proteins (dark gray). OM: outer membrane, PG: peptidoglycan, IM: inner membrane, TP: transpeptidase, TG: transglycosylase.

The divisome comprises over 30 proteins in *E. coli*, with at least 12 (e.g., FtsZ, ZipA, FtsQ, FtsB, FtsL…) being essential for survival (Figure 1) [10]. These proteins coordinate key stages of division: (i) Z-ring formation at the midcell, (ii) recruitment of division proteins, and (iii) PG synthesis and septation. In step (ii), the following late-stage proteins are recruited sequentially: FtsK → FtsQ → (FtsL, FtsB) → FtsW → FtsI → FtsN. These late proteins coordinate septal peptidoglycan synthesis and cell wall remodeling, which leads to divisome maturation and function [11]. The FtsQBL complex (comprising FtsQ, FtsB, and FtsL) serves as a critical bridge between early- and late-stage proteins, scaffolding the recruitment of additional components such as FtsW, FtsI, and FtsN. FtsW and FtsI are essential for PG synthesis, while FtsN acts as a trigger for this process, providing a feedback mechanism that ensures divisome assembly is complete before initiating septation [12,13]. This process is further fine-tuned by several regulatory, hydrolytic, and synthetic proteins that are not immediately subtle antibiotic targets because much of their activity is redundant and can be taken over by other proteins [14].

The divisome is an attractive target for novel antimicrobials, as disrupting its machinery halts bacterial cell division and propagation [15]. Among its components, the FtsQBL complex plays a critical role in cell division of G− bacteria [12]. Despite its essential role in divisome function and bacterial survival, the FtsQBL complex appears to be less extensively studied than other well-characterized targets like FtsZ and ZipA, making it a promising focus for innovative antibacterial strategies. Located in the periplasm, it offers unique advantages over intracellular targets exploited by conventional antibiotics. Inhibitors targeting this complex need only penetrate the outer membrane to access the periplasmic space, potentially bypassing challenges like cytoplasmic drug access and resistance mechanisms such as drug-efflux pumps [16]. Moreover, FtsQ, a key component of the complex, is present at exceptionally low cellular levels (20–300 copies per cell) in *E. coli*, meaning that even inhibitors with moderate potency could effectively destabilize the assembly and disrupt bacterial cell division [17]. The FtsQBL complex also exhibits high conservation across G− bacteria and lacks human homologs, highlighting its potential for broad-spectrum activity and target specificity [18].

Ten crystal structures of FtsQ-related proteins, including FtsQBL and FtsWIQBL complexes, resolved from *E. coli* [19], *Yersinia enterocolitica* [20], and *Pseudomonas aeruginosa* [21], respectively, have been reported to date (Table S1). These structures reveal critical interaction surfaces that play an important structural and functional role in the divisome and could be utilized in structure-based drug design. Although FtsQ also interacts with other proteins, such as FtsN [22], FtsI(PBP3) [23], FtsW [22], and FtsK [24] besides FtsB and FtsL, no X-ray structures of complexes with these proteins have been reported to date. This absence makes in silico analyses, such as the one presented here, particularly valuable and important.

**Figure 2:**
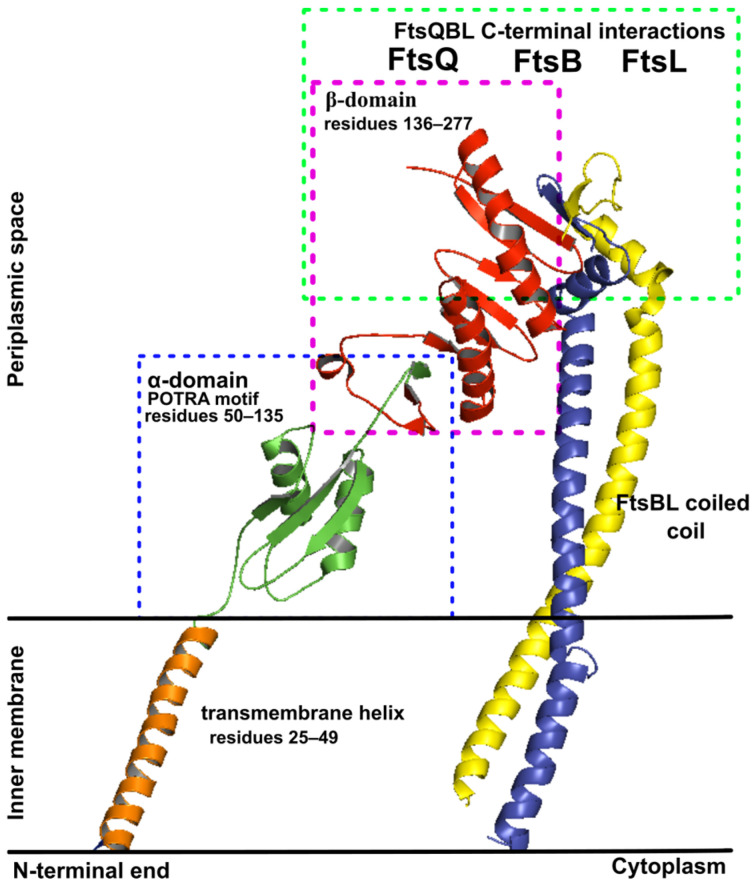
Structure of the FtsQBL complex (PDB ID 8HHG) [19].

The FtsQ protein in *E. coli* is a bitopic membrane rod-like shape protein characterized by a short *N*-terminal cytoplasmic domain, a transmembrane domain, and a larger periplasmic domain, which interacts with FtsBL at the C-terminal membrane-distal region and forms an inverted V-shape conformation (Figure 2) [19,20]. The periplasmic domain of FtsQ is essential both for FtsQ localization and for recruitment of FtsB and FtsL to the divisome [25,26]. It consists of two distinct globular subdomains named α and β, and a region that includes the last C-terminal 30 amino acids and is unstructured in the absence of other division proteins. α-domain, which resembles polypeptide transport-associated subdomain (POTRA), is located at the amino terminus of the periplasmic domain, whereas β-domain is located in the C-terminal part of FtsQ protein [10,27].

The primary interaction site between FtsQ and the FtsBL is located in the β-domain of FtsQ, which engages with a highly conserved 24-amino acid sequence in FtsB (Figure 2). This critical interface has been extensively characterized through structural and biochemical studies. The binding of FtsB to FtsQ demonstrates a submicromolar dissociation constant (~0.8 µM), as determined by surface plasmon resonance (SPR), highlighting the moderate strength and stability of the interaction, which is common for regulatory proteins [16,27].

Despite the significance of the FtsQBL complex as an AMR target, no potent small-molecule inhibitors have been developed to date. However, peptide-based compounds that mimic the structure of FtsB show promise, as they were shown to bind to the surface of FtsQ, resulting in potent antibacterial activity. In 2023, Paulussen et al. designed covalent macrocyclic inhibitors derived from FtsB’s periplasmic region, with an optimized inhibitor (K_D_ = 0.5 μM) disrupting the FtsQ-FtsB interaction, reducing bacterial growth in membrane-permeable *E. coli*, and improving survival in zebrafish infected with multidrug-resistant strains [28].

**Figure 3:**
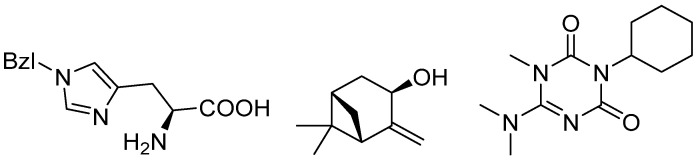
Hits from NMR screening of a fragment library showing low affinity (>1 mM) for the FtsQ interaction [29].

To date, evidence for the small-molecule ligandability of FtsQ remains limited. The only reported screening effort utilized NMR-based fragment screening of 1501 compounds against the periplasmic domain of FtsQ. This study identified only three fragments with low, millimolar affinity (Figure 3), which were not progressed further. Given that fragment-based approaches typically yield higher hit rates than standard drug-like libraries, the authors concluded that the FtsQ surface possesses low ligandability [29].

Although the role of FtsQ in bacterial cell division has been recognized for over 40 years, the lack of potent small-molecule modulators targeting the FtsQBL complex highlights the significant challenges in developing drugs for PPIs. Compared to classical drug targets like enzymes or receptors, there is no endogenous substrate for reference that a medicinal chemist could mimic. In addition, PPI interfaces are typically large (1200–2000 Å^2^), shallow, and hydrophobic, lacking the well-defined binding pockets commonly found in receptor-ligand interfaces, which reduces their ligandability [30,31]. Consequently, designing inhibitors that target these surfaces and comply with criteria like Lipinski’s Rule of 5 is challenging. That is why most molecules that target such surfaces have a higher molecular weight (>400 Da). These structural characteristics, combined with the relatively high affinity of binding between FtsQ and FtsB, make it difficult for small molecules to disrupt these interactions effectively [32,33].

Despite these challenges, the past decade has seen remarkable advancements in techniques facilitating the development of inhibitors for traditionally undruggable surfaces, exemplified by the increasing number of small-molecule PPI inhibitors, some of which are now in clinical trials [34]. A wide variety of strategies have been used to identify PPI modulator hits in recent years, including high-throughput screening, fragment-based drug discovery, and virtual screening. Although classical chemical libraries initially proved not to be suitable for screening against PPI surfaces, construction of some chemical libraries has been tailored to address the complexity of PPI interfaces also [35,36].

In addition, there have been many new findings about the nature of interactions that are formed between small molecules and PPI surfaces [37]. Small molecules interact with PPI interfaces through two primary mechanisms, orthosteric and allosteric, that can be further divided into subcategories [38]. Orthosteric modulators target specific, druggable regions on the PPI surface, preventing protein interactions. On the other hand, allosteric inhibitors attach to distant sites, triggering structural changes that weaken the binding affinity between proteins. Orthosteric modulators are ideal for PPIs featuring distinct hot-spot regions, whereas allosteric modulators are better suited for interfaces lacking clear pockets or those with flat surfaces. Certain modulators, termed molecular glues, enhance or stabilize PPIs by binding at the interface to create additional interaction points or by engaging allosteric sites to boost protein affinity, providing energetic benefits by reinforcing existing complexes [39,40]. On the other hand, ligands that target rim regions of the PPI surface modulate the interface by stabilizing or destabilizing protein interactions without directly competing with the core binding site. While most discovered modulators act as inhibitors, PPI stabilizers, especially molecular glues, hold significant potential due to their capacity to promote highly selective, non-native interactions [41,42,43,44,45].

One of the most critical aspects in designing small-molecule modulators targeting protein–protein interaction (PPI) surfaces is the identification of hot spots which contribute significantly to binding free energy (≥2.0 kcal/mol upon alanine mutation) and that make the design of small molecules even possible [46]. These residues form concave, druggable pockets (500–2000 Å^3^) characterized by a combination of hydrophobic and polar properties and are often enriched with tryptophan, arginine, and tyrosine [47]. Hotspots typically cluster at the center of the contact interface and are ideal for the design of orthosteric inhibitors or stabilizers. Only Y248, S250, and K59 have been identified as key hot spots on the FtsQ surface [19,26]. However, given the complexity of the interaction network involving FtsQ, it is likely that additional, yet-to-be-identified regions of the protein are still to be discovered.

Although experimental techniques such as X-ray crystallography, in vivo photo-crosslinking, and NMR-based fragment screening are widely used to identify PPI hotspots, they suffer from notable limitations [48]. These methods are often resource-intensive and limited by the solubility of probe molecules, and they frequently produce false positives by detecting fragments that bind to surface pockets not necessarily associated with PPI hot spots [49,50]. Moreover, while biochemically identified hotspots are critical for interactions with native protein partners, their suitability for small-molecule binding depends on favorable topological features, such as sufficient pocket concavity. Even when hot spots bind fragments with high affinity, they may still pose challenges as drug targets if they are part of small, isolated pockets that limit opportunities for fragment growing. Furthermore, even when hotspots bind fragments with high affinity, such sites may still be poor drug targets if they consist of shallow or isolated pockets that limit fragment growth and optimization. Therefore, only a limited subset of these hotspots qualifies as druggable sites for effective small-molecule drug binding [51].

To overcome these limitations and enhance data interpretability, it is essential to integrate experimental approaches with advanced computational methods [52]. Depending on the available data, computational strategies use different techniques to assess druggability. Roughly, they can be divided into ligand-based, precedence-based, structure-based, or sequence-based approaches. They are able not only to identify binding sites but also to assess their druggability during the target identification process [53]. However, a detailed description of each of these methods is beyond this manuscript, and for that I would refer the reader to some excellent reviews [52,54,55,56].

In this manuscript, we present a comprehensive computational analysis of the FtsQ protein to support the development of novel antibacterial therapeutics targeting AMR. Our objectives include analyzing the FtsQ protein surface and identifying potential binding sites using two established structure-based computational tools, FTMap [57] and SiteMap [58]. Both tools have been validated for analyzing PPI interfaces and demonstrate predictions of druggability that align well with X-ray crystallography and NMR data [48,59]. However, computational approaches often rely on predefined thresholds to classify binding pockets as druggable, which can lead to pockets being incorrectly identified as druggable [52]. To mitigate this limitation, we applied both techniques independently and integrated their results with analyses of evolutionary conservation and the physicochemical properties of the detected binding sites. Evolutionary conservation highlights residues of functional and structural importance, with highly conserved residues more likely to be characterized as hot spots [51,60]. Additionally, PPI interfaces are typically lipophilic, and binding sites lacking lipophilic residues are less likely to be druggable [61]. By combining these approaches, we aimed to enhance the accuracy of prioritizing druggable sites on FtsQ.

## 2. Results and Discussion

To analyze *E. coli* FtsQ as a potential antibacterial drug target, we performed a multi-level in silico analysis that included sequence conservation across Gram-negative pathogens, structure-based comparison of protein surfaces, evolutionary residue conservation, surface lipophilicity, conformational changes upon FtsB binding, and druggability of key interaction sites. These complementary approaches evaluated broad-spectrum potential, human off-target risks, and promising hotspots. The results are presented in the following subsections.

### 2.1. BLAST

We first examined the sequence conservation of *E. coli* FtsQ across clinically relevant Gram-negative bacteria using BLAST v2.17.0. The analysis focused on pathogens of particular clinical concern, defined by the KEGG pathogen database, including ESKAPE organisms [62] and species listed by the WHO as priority pathogens [63]. Our goal was to identify homologs of FtsQ, compare sequence similarity across species, and explore their evolutionary relationships through phylogenetic clustering. This allowed us to evaluate the potential of developing FtsQ inhibitors with a broad-spectrum activity and to estimate the applicability of our study to other clinically relevant bacteria.

**Figure 4:**
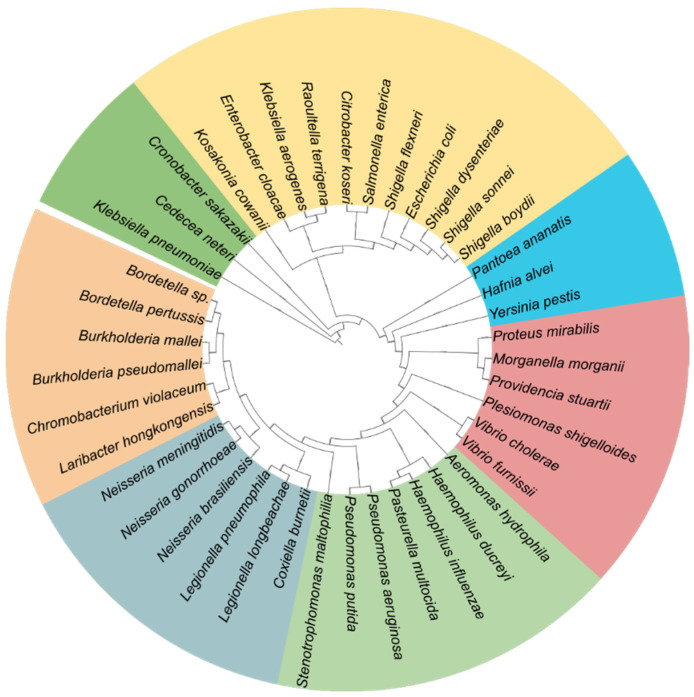
Phylogenetic tree of FtsQ homologs. Background colors highlight seven distinct phylogenetic clades. (e.g., *Enterobacteriaceae*, *Alcaligenaceae*/*Bordetella* group, etc.)

A phylogenetic tree was constructed based on multiple sequence alignments (MSA, Figure S1) of FtsQ homologs retrieved from the curated UniProtKB/Swiss-Prot database (Figure 4). Members of the *Enterobacteriaceae* family, including *Klebsiella*, *Enterobacter*, *Salmonella*, *Escherichia*, and *Shigella*, clustered closely together, indicating a high degree of FtsQ conservation within this group. In contrast, more divergent organisms, such as *Pseudomonas*, *Neisseria*, *Burkholderia*, and *Legionella* species, formed separate, distantly related clusters, consistent with their lower sequence identity to *E. coli* FtsQ. Quantitative analysis of representative sequence identity and positives revealed that *Shigella* species (*S. boydii*, *S. sonnei*, *S. dysenteriae*, and *S. flexneri*) exhibited the highest conservation, with 98.9–99.6% sequence identity and 99.3–100% positives (Table S2). High similarity was also predicted for *Salmonella enterica* (92.4% identity), *Klebsiella pneumoniae* (89.1%), *Klebsiella aerogenes* (87.8%), and *Enterobacter cloacae* (86.1%). Overall, the high level of conservation within these bacterial species suggests the potential feasibility of designing modulators capable of targeting multiple clinically important species within this group. In contrast, *Pseudomonas aeruginosa* and *Neisseria gonorrhoeae* displayed markedly lower similarity (31.9% and 34.7% identity, respectively), suggesting substantial divergence that may affect FtsQ structure or function. As a result, modulators targeting *E. coli* FtsQ are likely to have limited activity against these more distantly related organisms

Importantly, no eukaryotic proteins exhibited detectable similarity to FtsQ, which suggests a reduced likelihood of off-target effects in human hosts.

### 2.2. ProBiS

To evaluate FtsQ as a potential antibacterial drug target, we extended our sequence-based analysis with a structure-based comparison of protein structures. Although BLAST analysis showed that FtsQ lacks significant sequence similarity to eukaryotic proteins, sequence conservation alone does not rule out the possibility of structurally similar binding pockets. A drug can theoretically bind to human or bacterial proteins even with very low sequence or overall fold similarity [64]. Structure-based comparison, therefore, enables prediction of potential off-target interactions with human proteins or repurposing existing modulators of structurally similar proteins. We used the ProBiS web server [65] to compare the local 3D structure of *E. coli* FtsQ (PDB ID: 8HHG) [19] against structures in the Protein Data Bank that are not FtsQ. ProBiS compares protein surface regions based on their geometric and physicochemical properties, allowing detection of conserved binding sites even among proteins with low sequence similarity or divergent global folds. Z-scores, which quantify the statistical significance of local structural similarity, are also given as an output of ProBiS.

**Figure 5:**
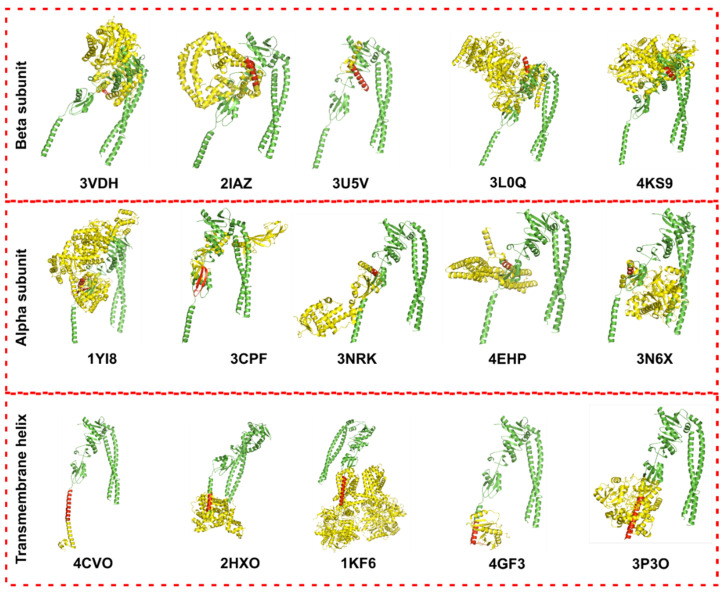
Superimposition of the top five proteins with the highest local structural similarity to the periplasmic domain of *E. coli* FtsQ (green; including transmembrane helix, α-domain, and β-domain), as identified by ProBiS. The similar proteins are shown in yellow, with the reference/aligned structure in red. PDB IDs are indicated.

The analysis focused on the transmembrane helix and the α- and β-subdomains of the cytosolic region of FtsQ, and the results are summarized in Figure 5 and Table S3. Overall, FtsQ has a very unique structure with relatively low Z-score similarity to known proteins, which reduces the risk of off-target effects in humans. Similarities were limited to small structural motifs, such as a single helix or loop. An exception was the eukaryotic translation initiation factor (PDB ID: 3CPF), where two antiparallel helices belonging to a conserved surface region in FtsQ’s α-subdomain (residues T86–V92 and S110–P116) displayed moderate structural similarity to a corresponding region of this protein. Consequently, modulators designed to target FtsQ could potentially exhibit off-target effects on eukaryotic cell regulation, although the limited extent of similarity (Z-Score = 1.8) suggests that such risks may be low.

Unfortunately, these low similarities also imply that repurposing existing modulators from similar proteins is unlikely to be effective for FtsQ. Similarly, machine learning approaches for drug-target interaction prediction [66], which often rely on similarities to known protein-ligand pairs, are less reliable for FtsQ due to its distinct binding sites. Consequently, classical drug design methods, such as molecular docking, are more suitable for developing FtsQ inhibitors.

### 2.3. Evolutionary Conservation

**Figure 6:**
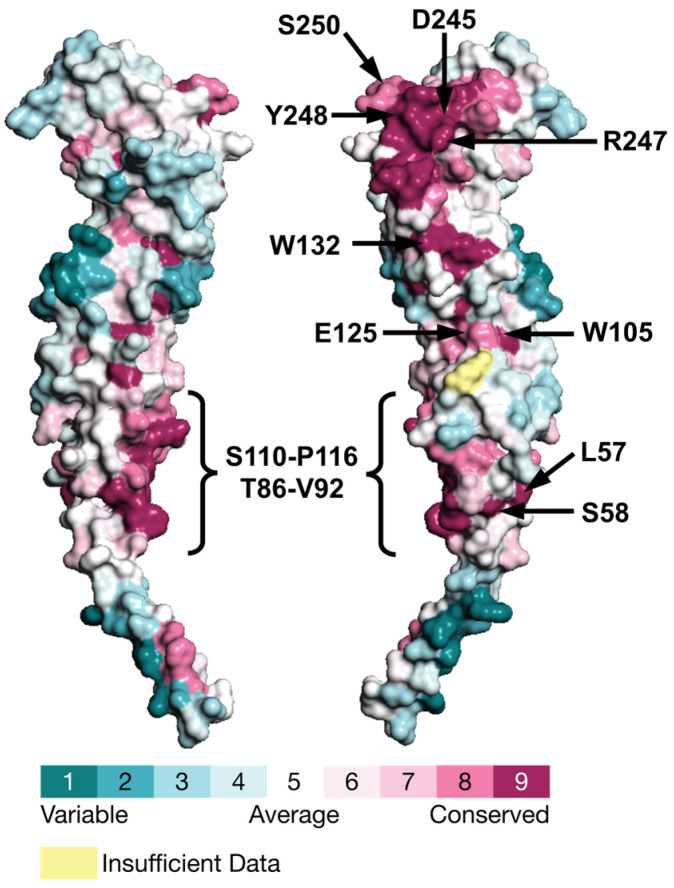
Surface representation of the FtsQ monomer (PDB ID: 8HHG [19]). Residues are colored by conservation using a turquoise-to-maroon gradient, from variable to highly conserved.

The evolutionary conservation of amino acid residues in a protein reflects a balance between their propensity for mutation and their essential role in preserving the protein’s structural stability and/or functional activity. Analyzing this conversation is therefore essential for pinpointing high-value targets in rational drug design. Therefore, we used the ConSurf web server [67] to map conservation scores (scores 1 to 9) onto the *E. coli* FtsQ structure (PDB ID: 8HHG) [19]. These scores are visually presented in Figure 6 (and Figure S2). Overall, FtsQ is moderately conserved, with 30% of residues having a conservation score of 8 or 9. The list of conserved residues in each domain, calculated based on amino acid frequency at each position, is presented in Table 1.

Mutational studies, combined with biochemical and biophysical techniques (e.g., surface plasmon resonance and site-specific photo-crosslinking), have identified several residues critical for protein function and demonstrated that disrupting these residues impairs cell division. ConSurf can complement these approaches by systematically mapping functional hotspots across the entire protein [67]. Unlike mutational studies, which target specific residues and may overlook broader evolutionary patterns, ConSurf analyzes sequence conservation across multiple species to identify evolutionarily constrained regions. This approach helps prioritize potential interaction sites or drug targets without requiring comprehensive experimental testing.

Overall, the POTRA and β-domain show higher conservation (36% and 30%, respectively) compared to the transmembrane helix (12% conserved) (Table 1). Conservation is not uniformly distributed, with certain regions of the POTRA and β-domain exhibiting a higher density of highly conserved residues. This suggests critical roles in structural stability or divisome interactions, as high conservation often correlates with functional importance.

Accordingly, residues at the interaction interface between the FtsQ and FtsB chains are mostly highly conserved (>65% conserved residues). This surface can be further divided into IS I (consisting of 8 conserved residues) and IS II (consisting of 6 conserved residues) (Table 1). Among these, residues D245, R247, and Y248 are 100% conserved, highlighting their critical importance. Our results align with mutational studies that identified Y248 as a key interaction hotspot. It was reported that the Y248W mutation disrupts FtsQ–FtsB association and causes severe cell division defects. We also predicted S250 as a conserved residue that was identified previously as an interaction hotspot [19,26].

The POTRA domain, which serves as a multifunctional hub for divisome assembly by interacting with early divisome proteins (FtsI, FtsN, FtsW), contains 30 conserved residues (Table 1). Evolutionary pressure on these residues likely stems from a complex network of divisome protein interactions, which complicates predictions of conservation patterns beyond direct binding roles. The lack of X-ray structures for FtsQ complexes with early divisome proteins makes mutational and conservation studies particularly valuable. Notably, the only 100% conserved residues, W105 and W132, are buried and likely stabilize the hydrophobic core of FtsQ. Our analysis predicted a conserved surface region (T86–V92 and S110–P116) that forms a hydrophobic pocket interacting with FtsI, FtsN, and FtsW through residues such as V92 and V111, as previously reported [20]. Other conserved residues are not part of any larger conserved area but have been identified by biochemical studies as interaction sites with FtsI, FtsN, and FtsL. For example, conserved residues P56, L57, and S58 form interactions with FtsI, while E125 interacts with FtsN [22,68].

### 2.4. Lipophilicity of FtsQ Surface

The strength of the interactions between two proteins or between a protein and an organic molecule is strongly influenced by the shape, size, and residue composition of the interaction site. Unlike enzyme drug-binding sites, PPI interfaces are larger, flatter, and rich in hydrophobic residues like threonine, alanine, and tyrosine [47,69,70,71]. Interactions at these interfaces are predominantly governed by hydrophobic contributions and further stabilized by favorable entropy gains, van der Waals interactions, and desolvation [72]. Additional interactions with arginine, which is also enriched at these surfaces, further enhance complex formation and stability through electrostatic and hydrogen bonds [73,74].

**Figure 7:**
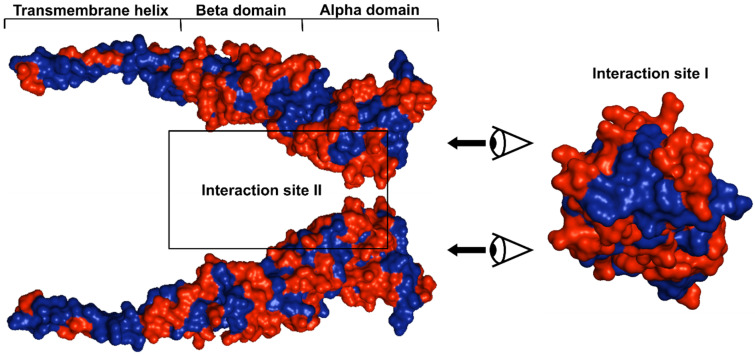
Surface representation of FtsQ showing hydrophobic and hydrophilic amino acids. Hydrophilic residues are colored red, and hydrophobic residues are colored blue.

Given the role of lipophilicity in druggability, we analyzed the surface properties of the FtsQ protein. The lipophilicity of FtsQ is illustrated in Figure 7, revealing extensive hydrophobic regions across the protein. Unsurprisingly, the most prominent hydrophobic surface is located in the transmembrane helix. In the POTRA domain, hydrophobic residues do not form large continuous patches. Instead, they appear repeatedly in a characteristic zebra-like pattern throughout the entire chain, specifically in the segments comprising residues 55–57, 60–62, 77–85, and 94–95. The only larger hydrophobic area exists at the interface between the POTRA and β-barrel domains, involving residues 126–130 (POTRA domain) and residues 147–149 and 156–163 (β-barrel domain). Although no crystal structures directly confirm the role of these hydrophobic regions in binding to other divisome proteins, their prominence suggests potential importance in PPI, consistent with the hydrophobic nature of PPI interfaces.

The most critical hydrophobic surface is located at the apex of the FtsQ β-barrel domain, where the interaction site between Q and B chain is situated. In IS I, a large hydrophobic area extends beyond the core interaction site, as illustrated in the top-down view in Figure 7. In contrast, IS II, where the β-helix of FtsB (residues 50–75) interacts with FtsQ, features a much smaller hydrophobic patch. Notably, the residues directly involved in FtsB binding within IS II are primarily hydrophilic, which makes this interface atypical compared to standard hydrophobic PPI [71].

### 2.5. Structural Changes upon FtsB Binding

**Figure 8:**
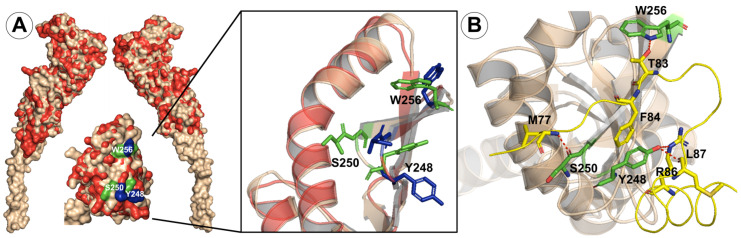
Conformational changes in FtsQ upon FtsB binding. (**A**) Overlay of FtsQ Complexed with FtsBL (PDB ID: 8HHG [19], red) and apo Form (PDB ID: 2VH1 [20], orange); (**B**) Interactions of FtsB chain with FtsQ chain in the ISI.

To develop effective small-molecule modulators targeting the FtsQ surface, it is critical to elucidate the conformational changes induced by FtsB binding to FtsQ. These changes can reveal transient binding sites and key interaction hot spots not apparent in the unbound protein, enabling the design of small molecules with enhanced specificity and affinity. With that in mind, we performed a comparison of the conformation of the Q chain in the FtsQBL complex (PDB ID: 8HHG [19]) with that in the apo FtsQ structure (PDB ID: 2VH1 [20]). Comparison of both X-ray structures, illustrated in Figure 8A, reveals minimal global conformational changes in the Q chain upon FtsB binding. However, significant local side-chain rearrangements occur in the Q chain, particularly in residues Y248, S250, and W256, which interact with FtsB. Specifically, Y248 engages in aromatic stacking with FtsB residue F84 and forms hydrogen bonds with FtsB R72 and M77 (Figure 8B). In addition, S250 and W256 form hydrogen bonds with L87 and T83, respectively, underscoring key interactions that stabilize the complex. These residue-specific shifts, especially with Y248 and S250, which were also identified experimentally as critical hot spots [19,26], indicate substantial surface remodeling at the interface between FtsQ and FtsB upon FtsB binding.

Although the interaction mode of the FtsB chain with the FtsQ surface differs from that expected for small-molecule binding, these structural insights are nonetheless pivotal for the rational design of small-molecule modulators targeting the FtsQB interface. For instance, the aromatic stacking and hydrogen-bonding interactions formed by FtsQ residue Y248 with FtsB residues F84, R72, and M77 create a distinct chemical microenvironment that small molecules could either mimic or selectively disrupt. Hot-spot residues such as Y248 and S250, which contribute substantially to binding affinity, therefore represent attractive targets for the development of tailored small-molecule scaffolds. While small molecules may, in principle, bind to apo or induced conformations of FtsQ, the apo structure does not capture the experimentally validated interface rearrangements observed upon FtsB binding, thereby increasing the uncertainty of docking predictions relative to FtsQB complex structures. Consequently, X-ray structures of FtsQB complexes are preferred over the apo form for structure-based docking studies.

### 2.6. Druggability

PPI modulators can be classified according to their mechanism of action as orthosteric or allosteric modulators, which may function either as inhibitors or stabilizers [43]. Orthosteric modulators, which are the most common class, can be further subdivided based on the location of their binding site into compounds that bind at the rim of the PPI interface and molecular glues that create additional interaction surfaces to stabilize the complex [41]. This mechanistic diversity prompted us to analyze various forms of the FtsQBL complex, including the FtsQ, FtsQB, FtsQL, and FtsQBL assemblies, to identify potential binding sites for various types of modulators. Analysis of the FtsQ surface in its uncomplexed form enables the identification of key hot spots and potential binding sites on the Q chain that may be targeted by small molecules. Such modulators could disrupt or stabilize interactions not only between FtsQ and FtsBL, but also with other components of this interaction network, such as FtsW, FtsN, or FtsI, for which no X-ray structures are currently available. Because binding of the B and L chains introduces additional potential binding surfaces on FtsQ, we included the full FtsQB and FtsQBL complexes in this analysis to explore opportunities for compounds that bind at the rim of multiple protein interfaces and stabilize the assembly. To avoid potentially misleading interpretations, assemblies containing only partial FtsB chains were excluded from the analysis.

The inclusion of the FtsQL complex is more controversial, as both X-ray structures and surface plasmon resonance data indicate that FtsL does not directly interact with FtsQ in the absence of FtsB [16,75,76]. Nevertheless, a ligand could theoretically bind to FtsQ and create an interface that facilitates FtsL association even in the absence of FtsB, resembling the mode of action of molecular glues. Interfacial inhibitors (or interfacial stabilizers), which modulate PPIs by binding to interfacial cavities, have long been underexplored as PPI targets. Nonetheless, several such modulators occur in natural products (e.g., fusicoccin, rapamycin, and brefeldin A). As a result, they have attracted increasing interest in recent years [38].

To increase the reliability of our analysis, we analyzed all seven available crystal structures of FtsQ from *E. coli* (Table S1) [16,19,20,27] and selected the one that exhibited the highest level of druggability, assuming that hypothetical ligands could be optimized to exploit the predicted hot spots. Although analyzing a larger ensemble of structures would be more accurate, the limited number of available structures made it impractical to combine these results and apply an additional threshold for druggability assessment as suggested in the literature [77]. Instead, the structure exhibiting the highest DScore was prioritized for in-depth characterization to establish a maximal druggable baseline for the protein.

To detect potential drug-binding regions on protein surfaces, we utilized two computational tools, FTMap [57] and SiteMap [58], both validated for identifying PPI interfaces [78,79]. Each program employs distinct algorithms, making them complementary in their approach. FTMap uses 16 small molecular probes to identify energetically favorable hotspots, which are generally less sensitive to conformational changes [80]. In contrast, SiteMap uses a grid-based approach to detect binding sites, which are larger and thus often include regions that may undergo conformational adjustments [80,81,82]. To avoid the risk of identifying functionally irrelevant pockets, we also considered their physicochemical properties, such as enclosure, size, and hydrophobicity, which are all calculated by SiteMap. In addition, we took into account evolutionary conservation calculated by ConSurf [67]. Highly conserved binding sites are more likely to elicit a response when modulated, as greater evolutionary conservation is associated with the functional and structural importance of residues in a protein. Nonetheless, this is not necessarily true for allosteric sites, which are typically much less conserved than regions directly important for the protein’s primary function. In fact, this lower degree of sequence conservation can facilitate the design of species-specific drugs [83].

#### 2.6.1. FTMap

The druggability of a PPI hotspot ensemble is determined by three key characteristics [77]: the strength of the primary hot spot (S, measured as cluster hit rate), the connectivity/compactness of the hot spot ensemble (CCD, measured as distance between cluster centers), and the maximum dimension of the ensemble (MD). These criteria allow binding sites to be classified as druggable, borderline druggable, or non-druggable based on their suitability for small molecule binding [80]. Detailed classification rules are provided in the Experimental Section.

In total, 180 binding sites were predicted across all crystal structures analyzed. Nevertheless, the majority of these sites (139) were classified as nondruggable, while a smaller subset (14 sites) that was deemed undruggable but featured a cluster in the vicinity of the ensemble that could potentially be exploited for binding, albeit likely with only high-micromolar potency. The primary reasons for the lack of druggability among the predicted hotspots were as follows: low S of primary hotspot (accounting for 88.5% of cases); pockets that were too shallow (MD < 10 Å) even when S exceeded 12 (10.8% of cases); or isolated hotspots with no opportunity to connect to secondary hotspots (CCD = 0, 11.5% of cases). Histograms with S, CCD, and MD distributions are shown in Figure S3.

**Figure 9:**
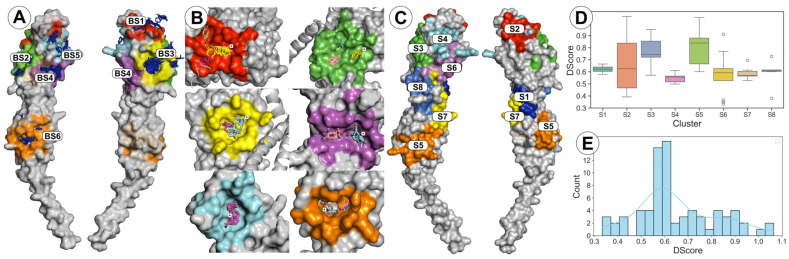
Binding sites on the FtsQ surface predicted by FTMap (**A**,**B**) and SiteMap (**C**–**E**). (**A**) All binding sites predicted by FTMap. (**B**) Top-ranked result for each binding site, with the primary hotspot annotated by an asterisk (*). (**C**) All binding sites predicted by SiteMap. (**D**) Boxplots showing the DScore distribution for each SiteMap binding site. (**E**) Histogram of the DScore distribution for all sites predicted by SiteMap. BS1–BS6 indicate binding sites predicted by FTMap; S1–S8 indicate sites predicted by SiteMap.

We detected 27 primary hotspots with druggability scores ranging from borderline druggable to druggable. These hotspots can be classified into six distinct potential binding sites, designated BS1 through BS6, and are mapped to the surface of FtsQ in Figure 9A. Close-up structural views of these sites are provided in Figure 9B. Data on the evolutionary conservation of residues in each respective site are shown in Figure S4C. Although BS2 was found to be undruggable based on geometry alone, we prioritized it for further study due to its significant evolutionary conservation and its later prediction as a druggable pocket by SiteMap. The S, CCD, and MD values, along with the predicted druggability class for the highest-ranked combination of crystal structure and complex form per binding site, are summarized in Table 2 and presented in more detail in Table S4. BS6 is the only site located in the POTRA domain, whereas the other five binding sites (BS1–BS5) are situated in the beta domain. This finding is unexpected, as the POTRA domain is known to interact with multiple proteins. In addition, our analysis of evolutionary conservation shows that a large portion of the POTRA domain is highly conserved, leading us to anticipate a higher number of binding sites in this region. The predicted binding sites exhibit variability in primary hotspot strength, evolutionary conservation, and lipophilicity, with detailed descriptions provided below.

#### 2.6.2. Sitemap

The druggability of a binding site depends on physicochemical properties such as pocket size, enclosure, and hydrophobic/hydrophilic balance. SiteMap identifies regions of high interaction potential and assigns a Druggability Score (DScore) using Equation (1) described in the Experimental Section. In addition, it also calculates several physicochemical parameters, including volume, enclosure, phobic, philic, and balance (Table 2 and Table S6). As detailed in the Experimental Section, we followed the druggability classification proposed by Alzyoud et al. [59], whereby sites were categorized based on DScore as very druggable (DScore ≥ 1.0), druggable (0.75 ≤ DScore < 1.0), moderately druggable (0.50 ≤ DScore < 0.75), or difficult (DScore < 0.50).

In total, 73 binding sites were predicted across all analyzed crystal structures (Table 2 and Table S6). Consistent with the FTMap results, the majority (62%) of these sites were predicted to be only moderately druggable. Overall, DScore values were relatively evenly distributed across the range of 0.3 to 1.1, as shown in Figure 9E. An exception was observed in the interval between 0.55 and 0.65, where a pronounced peak in frequency was detected. Notably, only 4% of all predicted binding sites were classified as druggable according to the SiteMap criteria. Comparison of physicochemical properties across the four druggability classes revealed that pocket volume and the polarity of surrounding residues are the strongest discriminants of druggability; these are also key input parameters for the calculation of the DScore (Table S5). Druggable sites exhibited larger pocket volumes (152–342 Å^3^) and higher hydrophobicity (Phobic = 0.885), reflecting favorable properties for small-molecule binding. In contrast, difficult sites displayed markedly lower volumes (64–83 Å^3^) and hydrophobicity (Phobic = 0.130), with a significantly reduced phobic/philic balance (0.094), indicating a predominance of polar and charged residues. Enclosure scores remained relatively consistent (0.59–0.71) across classes, suggesting that pocket shape and enclosure are less discriminatory than size and physicochemical composition in this dataset. Boxplots and histograms for the distribution of properties within all binding sites are provided in the Supporting Information (Figures S8 and S9).

In contrast to the heatmaps predicted by FTMap, where there was very little variability in the position of each hotspot between crystal structures, here the shape and surface of some binding sites varied substantially. This variability reflects the dynamic nature of the FtsQ surface, where local conformational reshaping directly impacts the predicted pocket depth and enclosure. To account for this variability and to map these 73 individual predictions into functionally relevant regions, the residues of each site were transformed into binary fingerprints and clustered using HDBSCAN on UMAP embeddings. This clustering allowed us to condense the heterogeneous set of 73 predictions into eight robust consensus clusters (DBCV = 0.944; S1–S8; see Experimental Section for full details), which are presented in Figure 9C and Figure S11, with the distribution of DScores for each binding site presented in boxplots in Figure 9D. DScore ranges (e.g., 0.60–1.05 for the POTRA site S5) illustrate that druggability is not a static property but varies with the protein’s conformational state. To prioritize the most viable starting points for drug design, we selected the ‘best-case scenario’ combination of crystal structure and complex form (highest DScore) for in-depth characterization (Table 2). Similarly to the FTMap results, only one cluster (S5) was located in the POTRA domain, and there was one binding site that spanned both the β and POTRA domains (S7); however, this site was detected only in the 8HHF monomer and is most likely a crystallographic artifact rather than a physiologically relevant site.

#### 2.6.3. Binding Sites Predicted by Both Methods

Next, to obtain a more robust and comprehensive evaluation of potential binding sites on the FtsQ protein, we combined and compared the results obtained from both methods. Because each approach has distinct strengths and limitations, their combination helps to prioritize more important sites, enhance overall confidence in predictions, and reduce the likelihood of identifying false-positive results. FTMap identifies energetically favorable hot spots, including those located in shallow or otherwise challenging regions, but it may ignore the full spatial context of larger pockets. In contrast, SiteMap provides detailed physicochemical characterization of binding pockets and is particularly effective at detecting larger or more enclosed sites, although it can miss shallow, solvent-exposed, or energetically subtle hot spots [57,73].

Three-dimensional structural representations of both methods are presented in Figure 9, whereas a heatmap showing the mapping of results from both methods onto the protein surface, combined with evolutionary conservation for each residue, is presented in Figure S12.

##### Interaction Site I

The first predicted binding site (BS1, S2) is located at the highly conserved apex interface between FtsQ and FtsB, designated as Interaction Site I. This region is critical for divisome assembly, as supported by biochemical studies showing that mutations or deletions of residues in this site severely impair bacterial growth [75]. Consequently, this site represents a high-priority candidate for small molecule exploration, particularly as it is structurally well-characterized and was predicted by both methods.

Nonetheless, FTMap analysis suggested that BS1 is intrinsically challenging for small, drug-like molecules. In the apo-FtsQ state, only a few sparse probe clusters were detected, resulting in a ‘non-canonically druggable–small’ (DS) classification. This is attributed to the unfavorable, relatively flat geometry of interaction site I, which restricts the formation of well-defined, deep hotspots. High-affinity modulation of this site would likely require peptides, macrocycles, stapled peptides, or charged compounds rather than conventional small molecules to achieve high-affinity modulation. The inclusion of the FtsL chain in the analysis moderately improved probe clustering by creating a shallow cavity at the FtsQ–FtsL interface, yielding a borderline-druggable classification (B). However, the primary hotspot remained weak, suggesting that drug-like compounds might achieve only micromolar affinities. Predicted non-bonded interactions involved the highly conserved residues L226, L230, A252, V254, and W256 of FtsQ, and E115 of FtsL, while hydrogen bonding is sparse and mainly involves A252, V254, and E115 (Figure S6). Notably, no significant probe interactions were observed with Y284, a residue previously identified as a hotspot, further underscoring the requirement for concave pocket geometry to facilitate small-molecule engagement.

SiteMap analysis predicted a significantly larger and better-defined binding site S1. This pocket involves 13 residues from the FtsQ chain and several from the FtsL chain. Druggability scores (DScore) exhibited substantial variability (0.39–1.06) depending on the specific structural complex. In FtsQL complexes (where FtsB is absent), SiteMap detected a substantial cavity (≈350 Å^3^), resulting in the highest observed DScore values. This space becomes almost entirely occluded in FtsQB or FtsQBL assemblies upon the binding of FtsB.

Conversely, the pockets predicted in the FtsQB and FtsQBL states are considerably smaller (≈80 Å^3^) and were classified as difficult to challenging by our metric. Despite their limited volume, these ‘rim’ pockets remain attractive for the design of interface modulators. Interestingly, these peripheral regions were not detected by FTMap, which is consistent with that algorithm’s focus on energetic hotspots rather than overall geometric enclosure.

A smaller pocket (51 Å^3^) was also predicted exclusively in the apo-FtsQ structure (PDB ID: 2VH1), centered on Trp256 with a high DScore (1.15). However, this feature disappears upon the assembly of FtsB or FtsL. Given the availability of only one apo-FtsQ structure, it remains unclear whether this pocket represents a transient binding site or a crystallographic artifact (Figure S13).

Overall, this site represents a biologically essential but structurally challenging target for classical small-molecule modulation. Achieving high-affinity binding across diverse conformational states appears unlikely with conventional compounds. However, the site is particularly amenable to larger, more elaborate scaffolds, such as macrocycles, which can exploit transiently open or partially occluded spaces. Although peptides face limitations like short half-life due to hydrolysis, these molecules carry the structural information necessary for binding to the target and thus have high target specificity and affinity [34]. This approach was exploited by Paulussen et al., who designed a macrocyclic inhibitor meant to mimic parts of the FtsB chain. Their inhibitor showed some activity, but it was weak until they eventually turned it into a covalent inhibitor [28].

##### Interaction Site II

The presence of the FtsB and FtsL chains divides the region around Interaction Site II into two distinct subregions: Interaction Site IIa (mapped to BS2 and S3) and Interaction Site IIb (mapped to BS3). Given the proximity of these subregions to FtsB, we hypothesize that ligands targeting these sites may act allosterically to influence FtsQB assembly or function as ‘rim’ modulators.

##### Interaction Site IIa

Interaction site IIa (BS2 and S3) comprises approximately 17 residues, including several highly conserved positions (R196, S198, W199, L211, G212, D245, and R247). In the uncomplexed FtsQ structure, FTMap predicted multiple weak probe clusters in this region, with additional clusters appearing upon formation of the FtsQBL complex. However, upon closer analysis, we determined that this region is predicted to be poorly suited for classical small molecules under current structural constraints, as all clusters are weak, with the strongest having a binding strength of 12, likely due to its relatively flat geometry and limited enclosure.

In contrast, SiteMap classified binding sites in this region as challenging to moderately druggable, with DScore values ranging from 0.57 to 0.95. Importantly, the site was detected only in the presence of FtsB and/or FtsL, while a single, shallow pocket was observed in the FtsQ-only structure. The fully developed pocket emerged exclusively in the FtsQB and the FtsQBL complexes, reaching volumes of up to 233 Å^3^ and DScores of up to 0.95 (moderately druggable). Thus, the presence of FtsB and FtsL creates an extended interfacial surface that significantly enhances the apparent druggability of this region, which was predicted only by SiteMap.

Ligands targeting this site would likely act as rim binders or allosteric modulators of the FtsQBL complex. Nevertheless, despite its size and the presence of conserved residues, the combined assessment of both methods indicates that the pocket’s flat topology and limited enclosure render it theoretically bindable but extremely challenging to target with high-affinity, drug-like small molecules. Although this region could, in principle, be considered for small-molecule modulator design, the expected potency would likely be low, consistent with FTMap’s classification of the site as non-druggable. It is more likely that this site (BS2) would function as a secondary binding site for a macrocyclic or extended ligand targeting BS1 in the proximity while simultaneously engaging this adjacent interfacial region.

##### Interaction Site IIb

Binding region BS3 was consistently predicted by FTMap to contain strong primary hotspots (binding strength > 16) across all analyzed protein forms, including uncomplexed FtsQ, indicating a persistent propensity for ligand interaction independent of complex formation. These clusters were strong but relatively small (MD ≈ 9.8 Å) and therefore have a potential not as a standalone druggable pocket, but as a hotspot-rich surface region that may function as an allosteric or rim binding site for small or fragment-like ligands.

The predicted clusters engaged predominantly in hydrophobic interactions with residues W199, T216, E169, R197, and R213, and formed hydrogen bonds primarily with T216 and W199 (Figure S6). The presence of charged residues (E169, R197, and R213) further suggests the potential for electrostatic interactions with ligands containing complementary charged or polar groups. However, despite these favorable interaction features, SiteMap did not identify an independent druggable pocket in this region. Only partial overlap with residues assigned to neighboring sites (BS2 and BS3) was observed. This region of protein, therefore, includes energetically favorable interaction hotspots that may contribute to ligand binding as predicted by FTMap but lack sufficient enclosure and pocket-like geometry required for classical SiteMap detection.

Given its spatial proximity to the FtsB and L chains, ligand binding at BS3 could extend toward the FtsB and FtsL chains, where additional FTMap hotspots were observed in the FtsQBL complex. Alternatively, effective engagement of this region may require larger ligands such as peptides, peptidomimetics, or macrocycles that can bridge BS3 with adjacent regions (BS1, BS2, or other surface patches) to achieve sufficient binding affinity and functional modulation.

##### Area at the Base of the β-Domain

We predicted a cleft at the base of the β-domain of FtsQ, adjacent to the POTRA domain, which was ranked by FTMap (BS4) as the most druggable site on the protein. Probe molecules at this interface bind mainly through hydrophobic contacts with residues Y174, L188, R175, G178, Q179, and F186, with additional hydrogen bonds involving Y174, L188, and G178 (Figure S6). The CCD values, which lie just below the 8 Å cutoff, indicate that the site could accommodate medium-sized to slightly larger drug-like compounds.

SiteMap (S6), however, assessed this region as difficult (DScore = 0.33) to moderately druggable (DScore = 0.91), with a median DScore of 0.59. This lower score reflects the shallow nature of the pocket, its small volume (median 62 Å^3^), and limited enclosure. The pocket is predominantly hydrophobic and is the smallest among the major predicted sites (median volume = 62 Å^3^), with small enclosure and contact scores. Residues forming this site show low conservation across homologues. Among approximately 22 residues that are present in this site, only P158 and L181 are conserved. Consistent with this, we found no reports suggesting that this region is directly involved in protein–protein interactions essential for FtsQ function.

Overall, despite FTMap’s optimistic prediction, the SiteMap analysis suggests that developing high-affinity ligands targeting this site alone would be challenging due to its limited depth and enclosure. Nonetheless, several additional binding sites are located in close proximity to this region, including Interaction site IIa. It is therefore possible that an inhibitor could be designed for this site and extend into neighboring pockets, thereby increasing binding surface area and improving affinity.

##### Other Sites in the Beta Domain

Four additional predicted binding sites on FtsQ were predicted, but have poor druggability and a lack of structural stability. Because of that, we do not consider them as high priority, and we will mention them only briefly. BS5 (S4) in the beta domain showed low druggability (DScore 0.50–0.61) and high residue polarity, while S7 and S8 had small volumes and were only predicted by SiteMap. Notably, S7 and S8 are shallow hydrophobic grooves at the POTRA–β-domain junction that were not detected upon FtsB/FtsL binding. This suggests that complex assembly induces long-range conformational changes that abolish these pockets, rendering them unlikely physiological targets. Furthermore, S7 was excluded as a likely crystallographic artifact found only in a single structure (8HHF).

##### POTRA Domain

The most consistently detected pocket (BS6, S5) is located in a highly conserved region of the POTRA domain and likely represents the interaction site between FtsQ and FtsW/FtsI/FtsN, which was reported by a two-hybrid screening [22,68]. This site was classified as druggable by both FTMap and SiteMap. FTMap predicted a relatively small but strong primary hotspot (17 clusters, MD = 9.1 Å) and a secondary hotspot (12 clusters) separated by 4 Å, indicating a compact site. In contrast, SiteMap delineated a larger binding region observed in the majority of X-ray structures, encompassing 23 residues, several of which are highly conserved (L57, S58, D91, V92, Q96, I121, and E125). The reported DScore range (0.60–1.05) highlights a degree of variability that is likely linked to the conformational dynamics of the POTRA domain. Consequently, this site should be viewed as a promising hypothesis for experimental validation via site-directed mutagenesis or fragment-based screening, rather than an unequivocally druggable interface in all physiological states. The pocket shows high enclosure (median 0.71) and a well-balanced hydrophobic/hydrophilic character (median 0.96). It contains two positively charged residues, K59 and R74, alongside substantial hydrophobic surfaces, suggesting that a viable ligand would likely require acidic or negatively charged groups to interact with K59 and R74, as well as hydrophobic fragments to engage the apolar regions.

### 2.7. Limitations and Future Directions

Although our multi-tool computational workflow provides a robust and orthogonal assessment of FtsQ druggability, several limitations inherent to the in silico nature of the study must be considered. First, the analysis relied on static crystal structures, which may not fully capture conformational dynamics present in vivo. While multiple complex states were examined to partially address structural variability, transient or cryptic pockets may remain undetected. Second, druggability scores assess the potential of a site to bind drug-like molecules, but they do not predict actual binding affinity or whether ligand binding will meaningfully modulate protein function. Third, although evolutionary conservation was used to estimate the functional importance of detected sites, conservation alone does not ensure that a drug-like molecule can bind with sufficient affinity or produce the desired modulatory effect.

Despite these inherent limitations, this study provides a rational framework to prioritize binding sites and substantially reduce the experimental search space. Given the low cost and scalability of computational approaches, the most efficient path forward is not the immediate resource-intensive experimental validation of every predicted site. Instead, we recommend performing a much cheaper and faster focused virtual screening or fragment-based design against prioritized pockets. This allows thousands of scaffolds to be examined virtually before committing wet-lab resources. High-confidence virtual hits should then progress to biophysical confirmation (e.g., SPR, MST, or NMR) and structural characterization (X-ray crystallography or cryo-EM). This strategic sequence preserves the predictive nature of our workflow and transforms high-potential candidate pockets into a structured roadmap for future experimental validation, effectively accelerating the development of FtsQ-targeted antimicrobials.

## 3. Materials and Methods

### 3.1. BLAST

To investigate the sequence similarity of the *E. coli* FtsQ protein to other proteins, a BLAST (Basic Local Alignment Search Tool) analysis was performed using the UniProt database. The amino acid sequence of *E. coli* FtsQ (UniProt accession: P06136) was retrieved from the UniProt website [84] and submitted to the BLAST algorithm (version 2.13.0) to identify homologous sequences. To ensure high-quality and non-redundant results, the search was conducted against the manually curated UniProtKB/Swiss-Prot database using default parameters, including an E-value threshold of 10. The top 1000 most similar sequences were exported in CSV format for downstream processing.

Data processing and analysis were performed using the KNIME Analytics Platform (version 4.7.2) [85]. Bacterial names in the dataset were standardized to include only the genus and species (e.g., *E. coli*). To focus the analysis on clinically relevant organisms, the dataset was filtered against a comprehensive list of human pathogenic bacteria retrieved from the Kyoto Encyclopedia of Genes and Genomes (KEGG; https://www.genome.jp/kegg/; accessed on 14 October 2025). To avoid bias from over-represented strains and to ensure a high-quality phylogenetic reconstruction, the dataset was refined to include only unique representative sequences for each pathogenic species. This was achieved using the KNIME GroupBy node to aggregate sequences by bacterial species. For each species, the percent identity and percent positives (including conservative substitutions) were calculated relative to *E. coli* FtsQ. Accession numbers were exported to a CSV file and subsequently used in Jalview for multiple sequence alignment (MSA) with MuscleWS in Jalview software v2.11.5.1 [86,87] using the default settings. After converting the alignment to FASTA file format, a phylogenetic tree was calculated based on the neighbor-joining method using similarity scores calculated by BLOcks SUbstitution Matrix 62 (BLOSUM 62), which measures the evolutionary relationship between each pair of sequences in the alignment [88]. After converting the phylogenetic tree to a Newick format file (.txt), the phylogenetic tree was subsequently used by the iTOL v7 web service to visualize the phylogenetic tree [89].

### 3.2. ProBiS

ProBiS (Protein Binding Sites) [65] is a web server and set of tools developed by the National Institute of Chemistry, Slovenia, for analyzing protein structures. It is used to identify 3D similarities in protein binding sites based on physicochemical properties, such as hydrophobicity and charge, rather than relying on global sequence or fold similarity. By querying the PDB or AlphaFold databases, ProBiS identifies proteins with similar binding sites and transfers known ligands from these structures to FtsQ, predicting potential druggable sites. We used FtsQ from the FtsQBL complex of *Escherichia coli* (PDB ID 8HHG [19]) to identify similar proteins. Results of the similarity search were downloaded and further processed in KNIME [85].

### 3.3. Consurf

The ConSurf server [67] was used to estimate the evolutionary conservation of amino acid positions in FtsQ from *E. coli* (PDB: 8HHG, chain Q [19]), based on phylogenetic relationships among homologous sequences. ConSurf employs an empirical Bayesian method to calculate evolutionary rates, which are projected onto the protein’s 3D structure. ConSurf performed a homolog search using the following parameters: homolog search algorithm with 1 iteration, an E-value cutoff of 0.0001, protein database selection, and a maximum of 150 homologous sequences with a maximal sequence identity of 97% and a minimal identity of 35%. The MSA was constructed using the default alignment method, and conservation scores were calculated using the empirical Bayesian method with the default substitution model.

Conservation scores, reflecting the relative evolutionary conservation at each sequence site, were mapped onto the X-ray structure of FtsQ (PDB: 8HHG [19]) These scores were divided into a discrete scale of nine grades for visualization: the most variable positions (grade 1, turquoise), intermediately conserved positions (grade 5, white), and the most conserved positions (grade 9, maroon). Results were downloaded as a .pse file containing 3D coordinates and conservation scores. Figures mapping conservation scores onto the protein structures were generated using PyMOL (Version 2.4.1) [90]. Additionally, a list of ConSurf grades was imported into KNIME [85] to extract residue names, conservation scores, and identity thresholds.

### 3.4. Visualization

Graphical representation of the three-dimensional structures of proteins was performed using PyMOL v2.4.1 [90]. A phylogenetic tree was constructed using iTOL v7 [89]. The graphical abstract was created by BioRender (BioRender, Toronto, Canada; https://biorender.com).

### 3.5. FTMap

FTMap (https://ftmap.bu.edu/serverhelp.php; accessed on 14 October 2025) is a computational tool that maps binding hotspots on macromolecules using Fourier transform-based docking. It docks hundreds of thousands of probe molecules from a diverse set of 16 probe types, designed to mimic organic solvents used in crystallographic soaking experiments. These probe types include acetamide, acetonitrile, acetone, acetaldehyde, methylamine, benzaldehyde, benzene, isobutanol, cyclohexane, *N*,*N*-dimethylformamide, dimethyl ether, ethanol, ethane, phenol, isopropanol, and urea. For each probe, the top 2000 poses are retained. Prior to mapping, all ligands and crystallographic water molecules are removed, and probes are distributed across the entire protein surface without assumptions about binding site locations. The poses are then minimized, clustered, and ranked based on the number of nonbonded contacts between the protein and probes. Regions binding multiple probe clusters are termed consensus sites (CSs), with the site containing the most probe clusters designated as the primary hotspot and others as secondary hotspots.

PDB IDs of all published X-ray structures of *E. coli* FtsQ, in apo form or in complex with FtsB, FtsL, or both, were submitted to the FTMap server for analysis. Results were downloaded as PyMOL session files (.pse) for visualization, accompanied by text files detailing cluster information, probe compositions, and hydrogen-bonded and nonbonded interactions, including precise residue contact counts. A Python v3.9 script was used to analyze each .pse file, calculating: (1) the strength (S) of the primary hotspot at the presumed binding site, defined as the number of probe clusters in the primary hotspot, (2) the center-to-center distance (CCD) between the primary and closest secondary hotspot, indicating connectivity and compactness, and (3) the maximum dimension (MD), the largest distance between any two probe cluster atoms in the hotspot ensemble.

Druggability was assessed using the metric defined by Kozakov et al. [80]. A binding site was classified as druggable (D) for conventional, drug-like compounds (e.g., rule-of-five-compliant small molecules) if it met the following criteria: (1) a strong primary hotspot with at least 16 probe clusters (S ≥ 16); (2) one or more secondary hotspots within a center-to-center distance (CCD) of less than 8 Å from the primary hotspot; and (3) a maximum dimension (MD) of the connected hotspot ensemble of at least 10 Å (MD ≥ 10 Å). Binding sites with a slightly weaker primary hotspot (13 ≤ S < 16) but meeting the CCD and MD criteria were classified as “borderline” druggable. Sites with a strong primary hotspot (S ≥ 16) and good connectivity (CCD < 8 Å) but a smaller ensemble dimension (7 Å ≤ MD < 10 Å) were considered non-canonically druggable—small (DS), indicating potential for binding by larger, charged, peptide-like, or macrocyclic compounds rather than traditional small molecules.

### 3.6. Sitemap

SiteMap (Schrödinger, Inc., New York, NY, USA) [58] was used to evaluate the druggability of potential binding sites on *Escherichia coli* FtsQ across multiple PDB structures representing four protein complexes: FtsQ, FtsQB, FtsQL, and FtsQBL. SiteMap identifies potential binding sites by calculating interaction energies between the protein and grid probes and by connecting “site points” that facilitate tight protein–ligand or protein–protein interactions. SiteMap calculates physicochemical attributes of each binding site, such as size, volume, enclosure/exposure, contact, hydrophobicity/hydrophilicity, balance of hydrophobic and hydrophilic character, and hydrogen-bonding (acceptor/donor) which are then used to quantify druggability using a Druggability Score (DScore), a composite metric that primarily rewards pocket size, degree of enclosure, and hydrophobic character while more heavily penalizing excessive hydrophilicity (relative to the related SiteScore).

The following equation is used to calculate DScore:

(1) $$\mathrm{Dscore}=0.094\sqrt{n}+0.60e-0.324p$$

where

n = the number of site points found for the site, capped at 100,
e = the degree of enclosure of the site
p = the hydrophilic score computed for the site.

Although threshold values for these parameters have been established in SiteMap [58] for typical small-molecule inhibitors (where a DScore of ≥ 0.8 is typically used as a threshold for druggable binding sites), these values are not valid for PPI interfaces, which often require higher hydrophobic character and larger surface areas. Therefore, we used the metric established by Alzyoud et al. [59], which categorized sites into four groups based on DScore: druggable (DScore ≥ 1.0), moderately druggable (DScore ≥ 0.75 and < 1.0), challenging (DScore ≥ 0.50 and < 0.75), and difficult (DScore < 0.50). These threshold values have been established and extensively validated on a set of 320 crystal structures representing 12 PPI targets.

All FtsQ X-ray structures (uncomplexed or in complex with FtsB and/or FtsL) were retrieved from the PDB and prepared in Maestro using the Protein Preparation Wizard. Water molecules and salts were removed, bond orders were automatically assigned, hydrogens were added, and missing side chains were built. Water molecules beyond a 5 Å radius of heteroatoms were reintroduced, and heteroatoms were protonated at pH 7.0. The impref script was used to perform constrained minimization of the protein with a maximum root mean square deviation (RMSD) of 0.30 Å for heavy atoms. One chain was retained from 2VH1; the partial B chain was removed from the FtsB complexes in 6H9N, 6H9O, and 5Z2W. FtsQ, in uncomplexed form or in complex with FtsB, FtsL, or both, was then analyzed using SiteMap. A minimum of 15 site points was required to define a binding site, and up to 15 sites were reported. A more restrictive definition of hydrophobicity was applied, and site maps were cropped at 4 Å from the nearest site points. Combined results were exported as SDF files for further analysis.

Data were processed in a Jupyter notebook. Only the following information about potential binding sites was retained: PDB_ID, Site, Complex, SiteScore, size, DScore, volume, exposure, enclosure, contact, phobic, philic, balance, don/acc, and residues. SiteMap also outputs the set of protein residues within a specified proximity (default 4.0 Å) of the predicted site for each binding site. Residues were renumbered using a Jalview alignment of all protein sequences with 8HHG-FtsQ [19] as the reference. Sites were encoded as binary sequence fingerprints (1 = residue present, 0 = absent). Sites with DScore < 0.50 were excluded to reduce noise, as they likely represent shallow surface features incapable of robust ligand stabilization. Because binding sites predicted by SiteMap were more heterogeneous than those from hotspot identification by FTMap, we did not classify each binding site manually. Instead, we used HDBSCAN combined with UMAP on Jaccard distances to define clusters of binding sites. HDBSCAN was chosen for its ability to detect variable-density clusters without a predefined number of clusters, while UMAP was selected because it preserves local and global structure better than t-SNE or PCA. Hyperparameters for HDBSCAN were tested in the following ranges: n_neighbors from 4 to 10, min_dist from 0.0 to 0.5, min_cluster_size from 2 to 6, and min_samples from 2 to 5. The optimal parameters (yielding maximum DBCV) were n_neighbors = 5, min_dist = 0.0, min_cluster_size = 4, and min_samples = 3, resulting in 8 clusters and a DBCV of 0.944. Results were exported in XLSX format. Finally, to ensure biological continuity, sites classified as ‘noise’ by the algorithm were subjected to expert structural inspection. Residues were reassigned to a cluster only if they were geometrically and structurally contiguous with a prioritized binding pocket.

To establish a baseline for the maximal druggable potential of FtsQ, we prioritized the structural state exhibiting the highest DScore for each site across the available ensemble. This ‘best-case scenario’ selection follows the methodology of Alzyoud et al. [59], who demonstrated that PPI interfaces are highly dynamic and that focusing on the most ‘open’ or ligand-amenable conformation is a necessary prioritization strategy in early-stage drug discovery. By targeting these high-tractability states, we aim to identify the structural features required for robust small-molecule or macrocyclic stabilization.

## 4. Conclusions

Computer-aided drug discovery methods provide valuable tools to study PPIs and characterize protein interfaces, especially when experimental data are limited. The main objective of this study was to assess FtsQ’s suitability as a drug target and provide guidance for structure-based design of novel modulators of divisome assembly. Overall, our results support FtsQ as a viable and challenging target for drug discovery. To our knowledge, this is the first systematic assessment of FtsQ’s conservation and druggability.

Our study combined multiple computational approaches, including evolutionary conservation analysis, sequence and structural comparisons across species, lipophilicity mapping, and druggability assessment. We found that our workflow is highly applicable to bacteria within the *Enterobacteriaceae* family, such as *Klebsiella pneumoniae* and *Enterobacter cloacae*, but its applicability decreases for more distantly related G− species, including *Pseudomonas aeruginosa* and *Neisseria gonorrhoeae*. From a drug design perspective, this highlights the potential to develop inhibitors tailored to closely related species. Notably, FtsQ exhibits negligible homology to eukaryotic proteins, which reduces the risk of off-target effects compared to FtsZ/FtsA inhibitors [91]. Conversely, the lack of closely related homologs with known modulators limits opportunities for ligand repurposing, favoring structure-based de novo strategies such as fragment-based design and docking-driven exploration of novel chemical space.

Although FtsQ’s surface lacks deeply buried canonical pockets, we generated a comprehensive map of its druggability landscape across multiple assembly states using probe-based hotspot detection (FTMap) and grid-based pocket characterization (SiteMap). Most predicted pockets exhibited moderate to low druggability, consistent with FtsQ’s central role in a dynamic PPI network and the general difficulty of targeting PPI interfaces with classical small molecules. A limited set of potentially ligandable sites was prioritized based on druggability metrics, physicochemical properties, and evolutionary conservation from ConSurf.

We predicted a previously undescribed pocket within the POTRA domain that represents a prioritized candidate site for conventional fragment- or structure-based drug discovery. This pocket is the only deep, well-defined, and enclosed site consistently predicted to be druggable. Its high evolutionary conservation, balanced hydrophobic/hydrophilic character, and strategically positioned positively charged residues make it particularly suitable for rational inhibitor design.

Another highly promising region is the conserved, hydrophobic FtsQB interface at the apex of FtsQ, essential for divisome assembly but structurally flat and thus challenging. FTMap detected weak or borderline hotspots, while SiteMap predicted transient pockets (up to ~350 Å^3^) primarily in FtsQL forms. High-affinity targeting with classical small molecules appears difficult. Larger modulators such as macrocycles, peptidomimetics, stapled peptides, or covalent inhibitors are more likely to achieve strong binding.

Additional potential binding sites for rim or allosteric modulators were predicted in Interaction Site IIb and Interaction Site I. While the latter are small and may have limited potency, IS IIb consistently showed strong ligand-binding potential, independent of complex formation. All other sites are considered low priority due to high polarity, shallow geometry, poor druggability, conformational instability, or potential artifacts.

Overall, our results characterize FtsQ as a viable but challenging target for drug discovery. However, the inherent limitations of a purely computational approach must be acknowledged. Although methods like FTMap and SiteMap are well-validated for identifying interaction hotspots, they rely on static or semi-flexible crystal structures that may not fully capture the extensive conformational plasticity of the divisome complex in vivo. Moreover, the provided druggability scores are predictive metrics of ligandability, not guarantees of biological efficacy.

Finally, this study establishes a systematic predictive framework to prioritize FtsQ’s most viable binding surfaces for future drug development. While our computational analysis suggests that predicted sites are likely to be functionally significant, they remain hypothetical until validated experimentally. Specifically, we propose targeting the POTRA interface with small molecules and the FtsQB site with macrocyclics. This structure-based roadmap, combined with experimental validation like phenotypic screening, provides a clear path toward new antibiotics for resistant pathogens. While many predicted surface pockets are poorly suited for classical small molecules, some may be amenable to alternative modulators such as macrocycles, peptidomimetics, or allosteric compounds.

## Figures and Tables

**Table 1:** Conserved (conservation score 8, black color) and highly conserved residues (conservation score 9, red color) in *E. coli* FtsQ motifs (PDB: 8HHG [19]), predicted by ConSurf [67]. Residues with 100% identity threshold are highlighted in bold red.

Motif	Residues
Transmembrane helix (res 25–49)	G27, F30, V34
IS I	L230, Y243, **Y248**, S250, G251, A253, V254, W256
IS II	R196, Q200, R213, R219, **D245**, **R247**
POTRA domain (residues)	P56, L57, S58, G64, T69, T86, F87, M88, Q90, D91, V92, Q96, P104, **W105**, I106, V109, S110, V111, R112, K113, Q114, W115, P116, L119, K120, I121, E125, A130, **W132**, N133
Beta subunit (residues)	D139, G142, **F145**, P158, L160, G162, P163, V170, L171, L181, M193, T194, R196, S198, W199, Q200, N205, L209, L211, **G212**, R213, R219, R222, F223, L230, Y243, **D245**, L246, **R247**, **Y248**, D249, S250, G251, A253, V254, G255, W256

**Table 2:** Classification of PPI binding sites based on druggability characteristics using FTMap and SiteMap. The highest-druggability binding sites were selected for each site.

FTMap					SiteMap						
BS ^a^	Complex	S	CCD (Å)	MD (Å)	BS ^b^	Complex	Dscore	Volume (Å^3^)	Enclosure	Balance	Phobic
					S1 ^MD^	FtsQBL	0.664	109	0.548	0.665	0.559
BS1 ^B^	FtsQL	15	5.8	14.7	S2 ^MD^	FtsQL	1.060	342	0.707	1.240	1.120
BS2 ^N^	FtsQB	12	7.4	13.0	S3 ^D^	FtsQB	0.950	233	0.495	1.070	0.944
BS3 ^DS^	FtsQBL	24	4.1	9.8							
BS4 ^D^	FtsQ	25	7.8	14.5	S6 ^D^	FtsQ	0.911	164	0.565	0.957	0.628
BS5 ^DS^	FtsQ	22	0	9.1	S4 ^MD^	FtsQ	0.567	125	0.618	0.910	0.094
BS6 ^DS^	FtsQL	17	4.4	9.1	S5 ^VD^	FtsQ	1.050	239	0.707	0.957	0.628
					S7 ^MD^*	FtsQ	0.695	79	0.512	1.830	0.760
					S8 ^MD^	FtsQ	0.727	83	0.773	0.594	0.708

^a^ FtMap druggability classes: ^D^—druggable, ^B^—borderline druggable, ^N^—nondruggable, ^DS^—Non-canonically druggable-small; ^b^ SiteMap druggability classes: ^D^—Druggable, ^MD^—Moderately druggable, ^VD^—Very druggable, ^MD^*—Moderately druggable, most likely a crystallographic artifact.

## Data Availability

The original contributions presented in this study are included in the article/Supplementary Material. Further inquiries can be directed to the corresponding author.

## Supplementary Materials

The following supporting information can be downloaded at: https://www.mdpi.com/article/10.3390/antibiotics15050430/s1, Figure S1: Multiple sequence alignment (MSA) of FtsQ from the clinically most relevant bacteria; Figure S2: Space-filling and surface representation of representative X-ray structures of FtsQ with degrees of conservation mapped onto the structure (ConSurf results); amino acids colored turquoise-through-maroon (variable-through-conserved); FtsB (blue) and FtsL (red) shown for comparison; Figure S3: Strength, cluster-to-cluster distance (CCD), and maximal dimension (MD) of cluster ensembles as identified by FTMap; Figure S4: Binding sites identified by FTMap; (a) all binding sites on the FtsQ surface; (b) best result for each binding site (primary hotspot annotated with asterisk *); (c) boxplot of ConSurf grades for residues surrounding each binding site; Figure S5: Lipophilic properties of each binding site identified by FTMap; lipophilic residues colored blue, hydrophilic residues colored red; Figure S6: Contact graphs displaying the contact rate (in %) of probes with surrounding residues for each binding site; Figure S7: Binding sites identified by SiteMap; (a) all binding sites on the FtsQ surface; (b) boxplot of DScores for each binding site; (c) DScore distribution of all binding sites; Figure S8: Distribution of features identified by SiteMap across all eight clusters; Figure S9: Distribution of features calculated by SiteMap between individual binding sites; Figure S10: Distribution of HDBSCAN clustering scores; Figure S11: 2D UMAP projection (HDBSCAN clusters); Figure S12: Druggability and evolutionary conservation of amino acid residues identified by FTMap, SiteMap, and ConSurf, presented as a heatmap; normalized scores shown for FTMap classification, SiteMap DScore, or normalized ConSurf score; numbers indicate corresponding FTMap binding sites (BS1–BS6) and SiteMap clusters (S1–S8); Figure S13: Comparison of binding sites identified in different complex forms of the FtsQBL complex by SiteMap; each label under the structure represents PDB ID and complex composition. Table S1: X-ray crystal structures of FtsQ protein; Table S2: Sequence similarity of FtsQ homologs from clinically relevant Gram-negative pathogens to *E. coli* FtsQ; Table S3: Top five most structurally similar proteins to the transmembrane helix, α-subunit, and β-subunit of FtsQ, as identified by ProBiS; Table S4: Binding sites identified by FTMap, including strength, cluster-to-cluster distance (CCD), maximal dimension (MD), and druggability classification; Table S5: Druggability classification of the 73 binding sites identified by SiteMap, showing the number of sites per class and median (range) values for DScore, pocket volume (Å^3^), hydrophobicity (phobic), hydrophobic/hydrophilic balance, and enclosure; Table S6: SiteMap results for each binding site in clusters S1–S8 (size, SiteScore, DScore, volume (Å^3^), exposure, enclosure, contact, hydrophobic (phobic) and hydrophilic (philic) properties, balance, and donor/acceptor ratio (don/acc)); Table S7: DScore values for all sites identified by SiteMap across different PDB structures and complexes. Ref. [92] is cited in Supplementary Materials.

## Abbreviations

The following abbreviations are used in this manuscript:

Abbreviation	Full Term
AMR	Antimicrobial resistance
BLAST	Basic Local Alignment Search Tool
BS	Binding site
CCD	Center-to-center distance
DScore	Druggability Score
FTMap	Fourier Transform Mapping
HDBSCAN	Hierarchical Density-Based Spatial Clustering of Applications with Noise
IM	Inner membrane
IS	Interaction site
KNIME	KNIME Analytics Platform
MD	Maximum dimension
MSA	Multiple sequence alignment
OM	Outer membrane
PDB	Protein Data Bank
PG	Peptidoglycan
PPI	Protein–protein interaction
POTRA	Polypeptide transport-associated
ProBiS	Protein Binding Sites
S	SiteMap binding site
SPR	Surface plasmon resonance
TP	Transpeptidase
TG	Transglycosylase
UniProt	Universal Protein Resource
WHO	World Health Organization
