# Supplementary material for: In Silico Druggability Assessment of Escherichia coli FtsQ Reveals Tractable PPI Interfaces in the Divisome
Source: Antibiotics (Basel). 2026 Apr 24;15(5):430. doi: 10.3390/antibiotics15050430 (PMC13203102; doi:10.3390/antibiotics15050430)
Supplement: Supplementary file 1 [file antibiotics-15-00430-s001.zip › antibiotics-4164510-supplementary.pdf]

Supporting information

In Silico Druggability Assessment of Escherichia coli FtsQ Reveals Tractable PPI Interfaces in the Divisome

Rok Frlan
The Department of Pharmaceutical Chemistry. Faculty of Pharmacy. University of Ljubljana. 1000 Ljubljana. Slovenia; rok.frlan@ffa.uni-lj.si;
Tel: +386-1-4769-674

Contents

1. Crystal structures 2
2. BLAST 2
3. Probis 3
4. ConSurf 4
5. FTMap 5
6. Sitemap 9
7. Comparisson FTMap vs Sitemap 17

1. Crystal structures

Table S1. X-ray crystal structures of FtsQ protein.

PDB ID	Organism	Complex	Chains	Res (Å)	Year [ref]
**2VH2**	*Yersinia enterocolitica*	FtsQ	A	3.6	2007 [1]
**2VH1**	*Escherichia coli*	FtsQ	A	4.2	2007 [1]
**6H9O**	*Escherichia coli K-12*	FtsQB (partial B)	A.B. C.D	2.94	2018 [2]
**5Z2W**	*Escherichia coli*	FtsQB (partial B)	A.B	3.26	2018 [3]
**8HHG**	*Escherichia coli K-12*	FtsQBL	Q.B.L	4.24	2022 [4]
**6H9N**	*Escherichia coli K-12*	FtsQB (partial B)	A.B	3.1	2018 [2]
**8HHH**	*Escherichia coli K-12*	FtsQBL	Q.B.L	4.31	2022 [4]
**8HHF**	*Escherichia coli K-12*	FtsQBL	Q.B.L	4.29	2022 [4]
**8BH1**	*Pseudomonas aeruginosa PAO1*	FtsWIQBL	A. B. C. D. E	3.8	2022 [5]
**8P1U**	*Pseudomonas aeruginosa*	FtsWIQBL	A. B. C. D. E	3.3	2023 [6]

2. BLAST

Table S2. Sequence similarity of FtsQ homologs from clinically relevant Gram-negative pathogens to *Escherichia coli* FtsQ.

Genus	Organism	UniprotID	Identity (%)	Positives (%)
Shigella	*Shigella boydii*	B2U298	99.6	100
Shigella	*Shigella sonnei*	Q3Z5R6	99.6	100
Shigella	*Shigella dysenteriae*	Q32JZ9	99.3	99.6
Shigella	*Shigella flexneri*	A0A0H2VSP0	98.9	99.3
Salmonella	*Salmonella enterica*	A0A4Z0NSV4	92.4	95.3
Klebsiella	*Klebsiella pneumoniae*	A0A378C1B7	89.1	93.7
Enterobacter	*Enterobacter cloacae*	A0A0H3CF00	86.1	91.1
Proteus	*Proteus mirabilis*	B4F108	61.9	75.8
Neisseria	*Neisseria gonorrhoeae*	Q5F6M1	34.7	54.8
Pseudomonas	*Pseudomonas aeruginosa*	G3XDA7	31.9	56

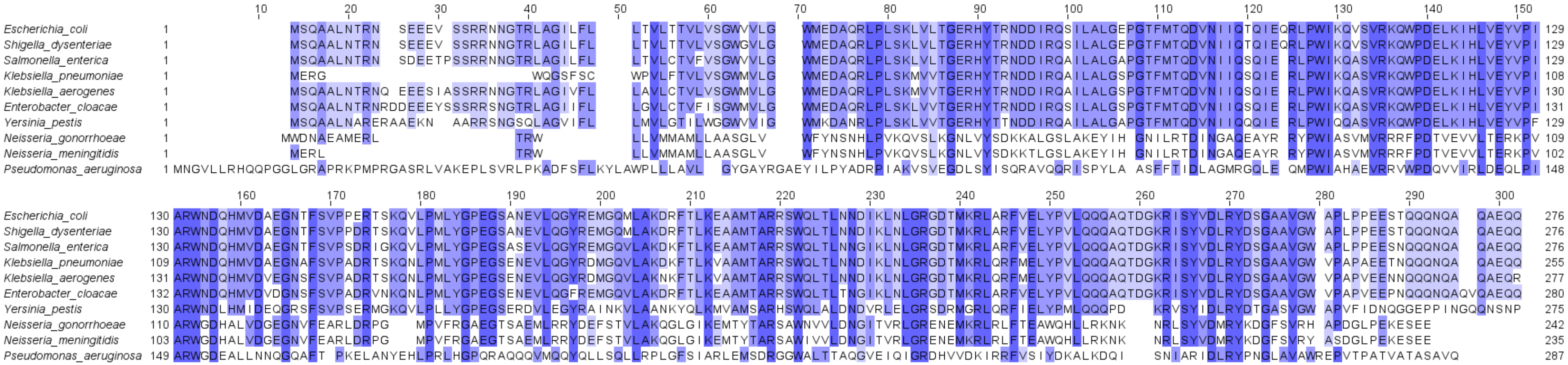

Figure S1. Multiple sequence alignment (MSA) of FtsQ from the clinically most relevant bacteria.

3. Probis

Table S3. Top five most structurally similar proteins to the transmembrane helix, α-subunit, and β-subunit of FtsQ, as identified by ProBiS.

PDB ID	Chain ID	Protein Name	Z-Score	Subunit
**3n6x**	A	Putative glutathionyl spermidine synthase	2.08	Alfa
**4ehp**	B	Catenin alpha-1	1.85	Alfa
**1yi8**	A	Tryptophanyl-trna synthetase	1.84	Alfa
**3cpf**	A	Eukaryotic translation initiation factor 5a-1	1.8	Alfa
**3nrk**	A	Lic12922	1.79	Alfa
**3vdh**	A	β-1.4-endoglucanase	2.1	Beta
**2iaz**	A	Hypothetical protein sp1372	2.01	Beta
**3u5v**	A	Protein max. transcription factor e2-alpha chimera	1.95	Beta
**3l0q**	A	Xylulose kinase	1.92	Beta
**4ks9**	A	Malonyl-CoA decarboxylase	1.91	Beta
**4cvo**	A	DNA excision repair protein ercc-6	2.16	Membrana
**2hxo**	A	Putative tetr-family transcriptional regulator	2.14	Membrana
**1kf6**	C	Fumarate reductase 15 kda hydrophobic protein	2.08	Membrana

**4gf3**	B	Tyrosine-protein phosphatase yoph	2.06	Membrana
**3p3o**	A	Cytochrome P450	2.05	Membrana

4. ConSurf

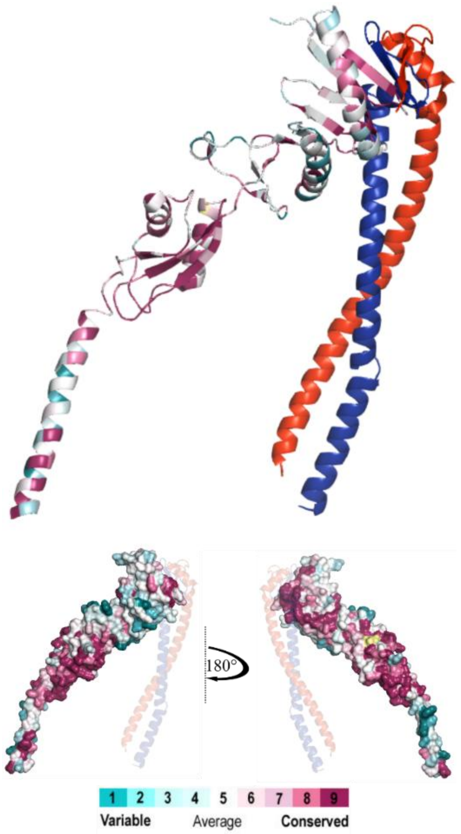

Figure S2. Spacefill and surface representation of the representative X-ray structures of FtsQ with the evolutionary conservation mapped onto the structure. Amino acids are colored according to their degree of conservation using the color-coding bar with turquoise-through-maroon indicating variable-through-conserved residues. FtsB (Blue) and FstL (red) are also presented for a comparison.

5. FTMap

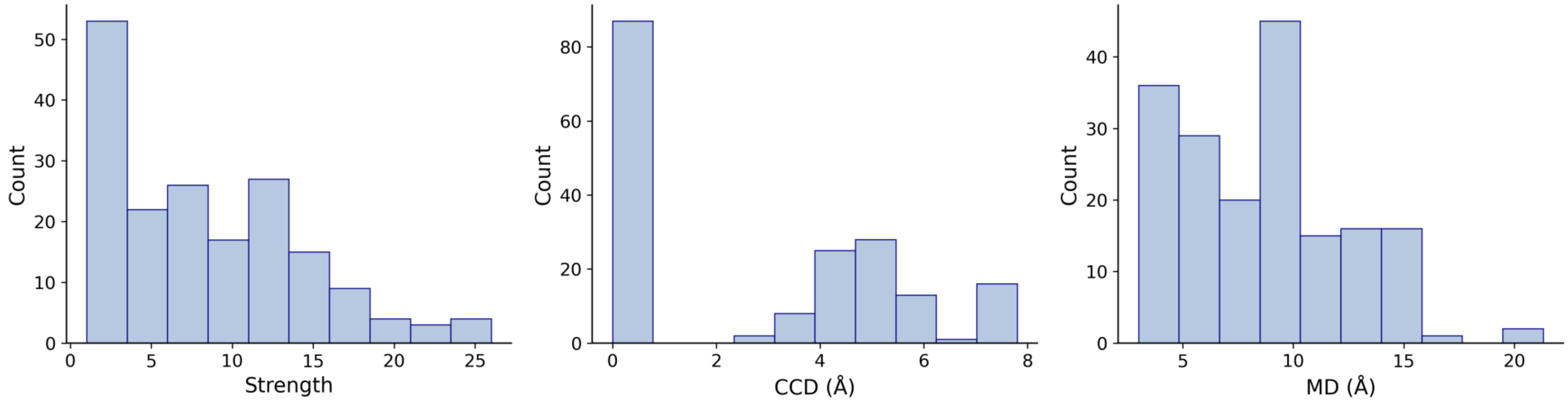

Figure S3. Strength cluster-to-cluster distance (CCD) and maximal dimention (MD) of cluster ensambels as identified by FTMap.

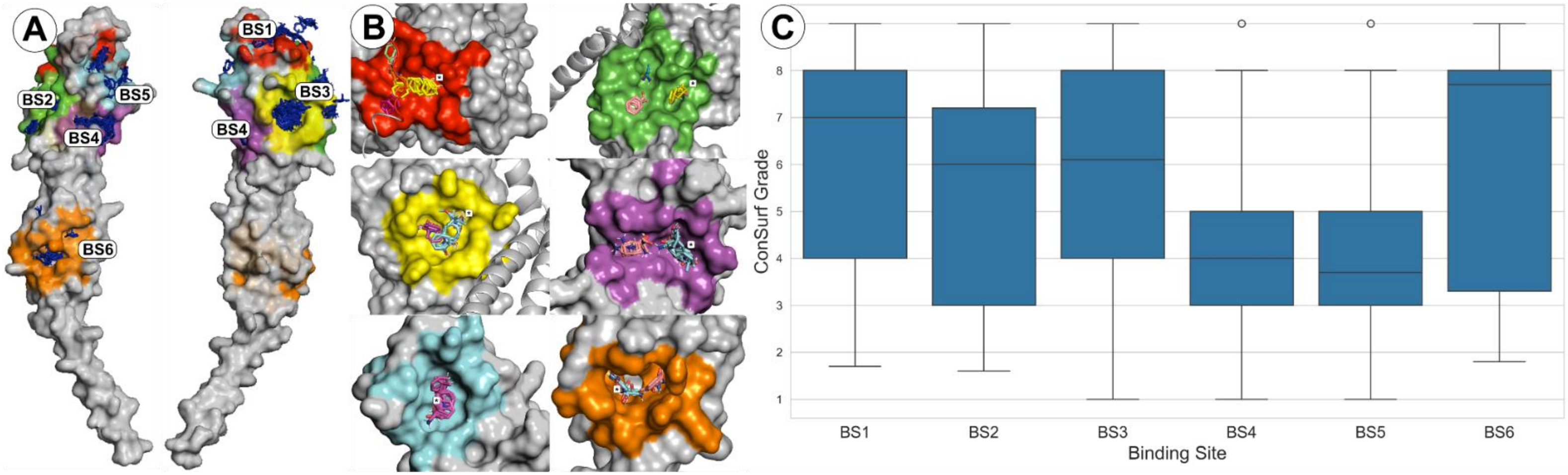

Figure S4. Binding sites identified by FtMap. (**a**) Presentation of all binding sites on FtsQ surface. (**b**) Best result for each binding site (primary hotspot annotated with asterisk*; (**c**) Boxplot of ConSurf grades for residues surrounding each binding site.

Table S4. Binding sites identified by FTMap, including strength, cluster-to-cluster distance (CCD), maximal dimension (MD), and druggability classification.

Binding_Site	PDB_ID	Complex	Strength	CCD	MD	Druggability_Class*
**BS1**	2VH1	FtsQ	19	3	9,7	DS
**BS1**	6H9N	FtsQ	19	4	9,4	DS
**BS1**	8HHG	FtsQL	13	4,7	15	B*
**BS1**	8HHH	FtsQL	15	5,8	15	B*
**BS2**	8HHH	FtsQB	12	7,4	13	N*
**BS2**	8HHH	FtsQB	11	7,1	13	N*
**BS2**	8HHH	FtsQBL	11	7,3	15	N*
**BS3**	8HHF	FtsQB	15	5,2	9,7	BS
**BS3**	8HHF	FtsQBL	15	4,6	9,7	BS
**BS3**	8HHF	FtsQBL	16	4,6	9,7	DS
**BS3**	8HHG	FtsQ	19	4,8	9,5	DS
**BS3**	8HHG	FtsQB	15	4,7	12	B
**BS3**	8HHG	FtsQB	18	4,7	12	D
**BS3**	8HHG	FtsQBL	19	4,9	12	D
**BS3**	8HHG	FtsQBL	15	4,9	12	B
**BS3**	8HHG	FtsQL	15	4,1	9,7	BS
**BS3**	8HHH	FtsQ	18	4	9,3	DS
**BS3**	8HHH	FtsQB	14	4,1	9,8	BS
**BS3**	8HHH	FtsQBL	24	4,1	9,8	DS
**BS3**	8HHH	FtsQBL	15	4,1	9,8	BS
**BS4**	8HHF	FtsQ	16	7,3	14	D
**BS4**	8HHG	FtsQ	23	7,2	15	D
**BS4**	8HHG	FtsQ	13	7,2	15	B
**BS4**	8HHH	FtsQ	14	3,4	13	B
**BS4**	8HHH	FtsQ	10	4,9	13	N*
**BS5**	2VH1	FtsQ	15	4	13	B
**BS5**	6H9O	FtsQ	25	7,8	15	D
**BS6**	8HHF	FtsQ	13	5,1	9,1	BS
**BS6**	8HHF	FtsQ	17	5,1	9,1	DS

**BS6**	8HHF	FtsQB	14	4,9	9,2	BS
**BS6**	8HHF	FtsQL	17	4,4	9,1	DS

*D-druggable, B-borderline druggable, N-nondruggable, DS-Non-canonically druggable–small.

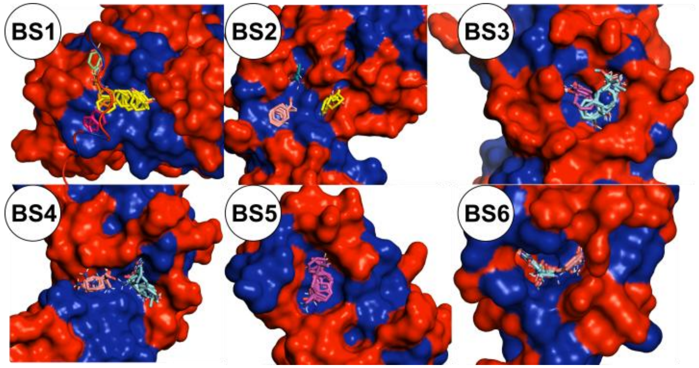

Figure S5. Lipophilic properties of each binding site identified by FTMap. Lipophilic residues are colored blue; hydrophylic residues are colored red.

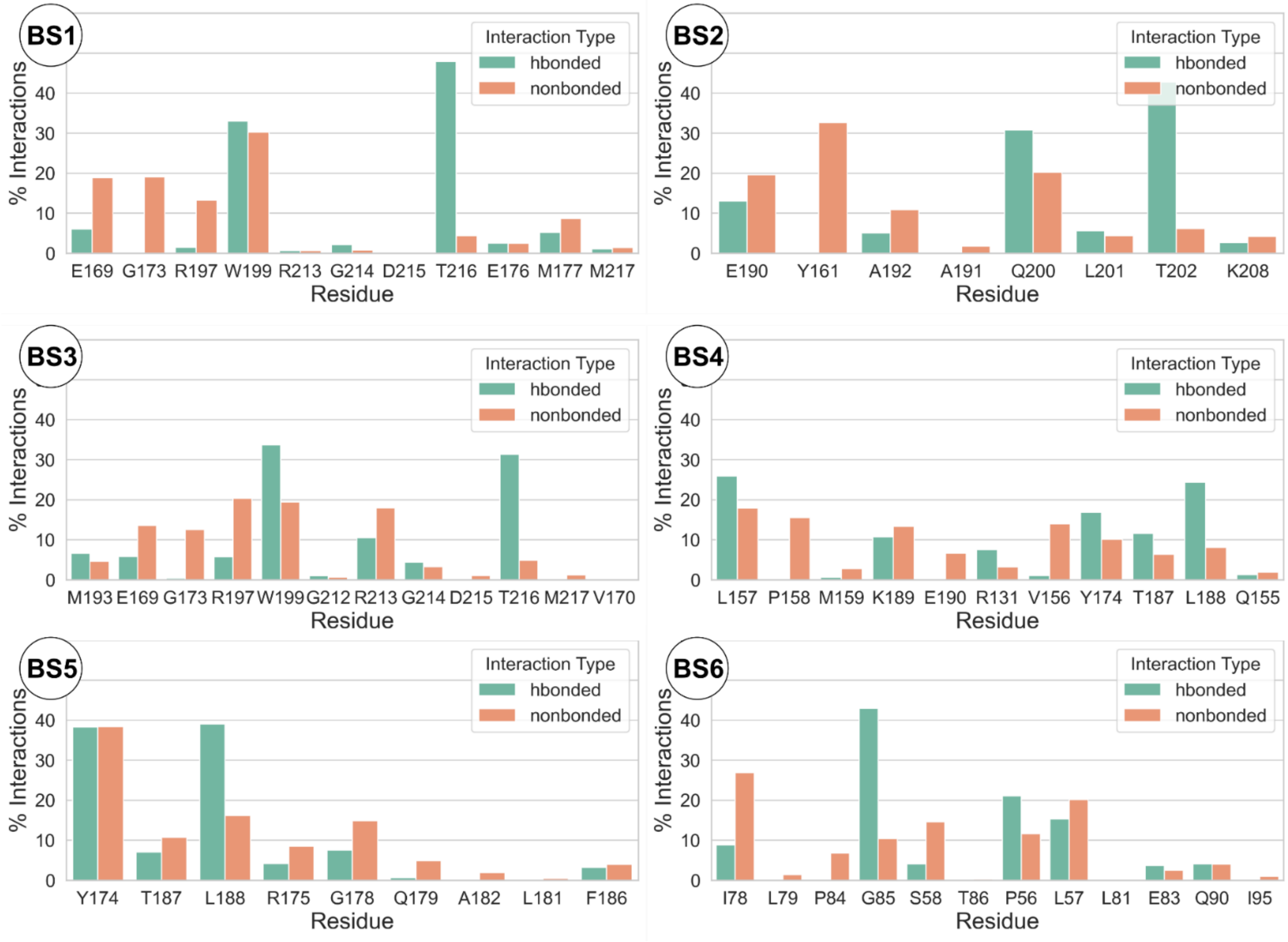

Figure S6. Contact graphs displaying the contact rate (in %) of probes with surrounding residues for each binding site.

6. Sitemap

Table S5. Druggability classification of the 73 binding sites identified by SiteMap, showing the number of sites per class and median (range) values for DScore, pocket volume (Å³), hydrophobicity (phobic), hydrophobicity/hydrophilicity balance, and enclosure.

Druggability	No.	Dscore (range)	Volume/Å³ (range)	Phobic (range)	Balance (range)	Enclosure (range)
Druggable	3	1.05 (0.050)	342.0 (137.0)	0.885 (0.612)	0.91 (0.680)	0.707 (0.102)
Moderately druggable	15	0.873 (0.179)	152.0 (331.1)	0.764 (2.026)	0.933 (2.768)	0.673 (0.329)
Challenging	45	0.602 (0.245)	83.3 (148.2)	0.559 (1.781)	0.665 (3.21)	0.593 (0.261)
Difficult	10	0.403 (0.164)	64.5 (60.1)	0.131 (0.335)	0.094 (0.354)	0.605 (0.289)

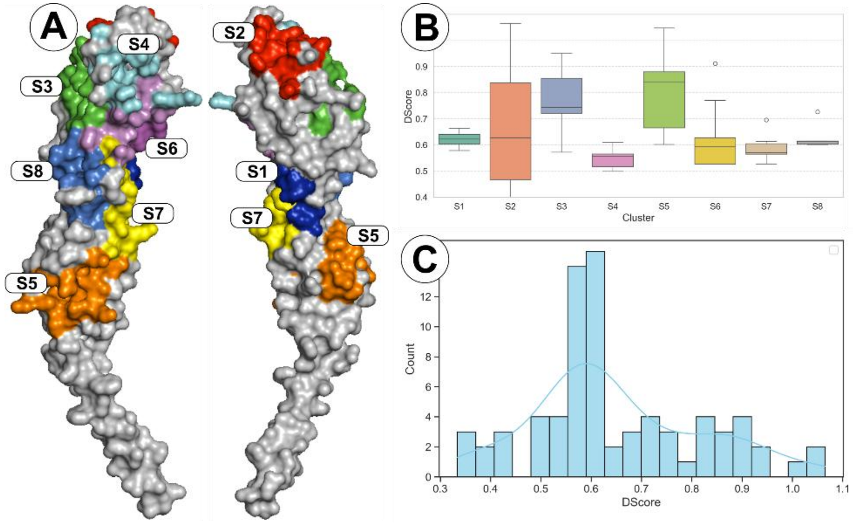

Figure S7. Binding sites identified by SiteMap. (**a**) Presentation of all binding sites on FtsQ surface. (**b**) Boxplot of DScores for each binding site. (**c**) DScore distribution of all binding sites.

Table S6. SiteMap results for each binding site in clusters S1-S8. Size, SiteScore, Dscore, Volume (Å3), exposure, enclosure, contact, hydrophobic (phobic) and hydrophilic (philic) properties, balance and ratio between proton donnor and acceptor properties of each ligand (don/acc) are given.

PDB ID	Complex	cluster	Site	size	SiteScore	Dscore	Volume (Å^3)	exposure	enclosure	contact	phobic	philic	balance	don/acc
8HHF	FtsQ	S1	4	33	0.612	0.578	104	0.752	0.533	0.694	0.577	0.86	0.668	0.916
8HHF	FtsQB	S1	2	37	0.639	0.612	102	0.76	0.538	0.717	0.52	0.87	0.599	0.884
8HHF	FtsQBL	S1	2	42	0.68	0.664	109	0.725	0.548	0.715	0.559	0.84	0.665	0.931
8HHF	FtsQL	S1	4	38	0.646	0.632	104	0.753	0.519	0.680	0.63	0.80	0.792	1.070
2VH1	FtsQ	S2	1	32	0.714	0.716	50.8	0.536	0.603	0.828	1.52	0.55	2.800	2.480
8HHG	FtsQB	S2	3	45	0.68	0.545	93.6	0.664	0.580	0.768	0.201	1.33	0.151	0.280
8HHH	FtsQB	S2	4	80	0.8	0.669	86.1	0.717	0.515	0.768	0.029	1.47	0.020	0.880
8HHF	FtsQB	S2	6	25	0.614	0.414	66.2	0.615	0.669	0.930	0.204	1.40	0.146	0.481
8HHH	FtsQB	S2	7	40	0.555	0.392	32.9	0.791	0.436	0.798	0.029	1.42	0.020	0.994
8HHG	FtsQBL	S2	5	25	0.642	0.583	82.7	0.675	0.664	0.853	0.574	0.88	0.655	0.713
8HHF	FtsQBL	S2	5	27	0.666	0.418	77.9	0.550	0.725	1.070	0.206	1.55	0.133	0.488
8HHH	FtsQBL	S2	4	23	0.551	0.482	84.4	0.742	0.575	0.662	0.352	0.96	0.366	0.748
8HHF	FtsQL	S2	1	127	0.963	1.01	376	0.666	0.604	0.783	0.508	0.91	0.559	0.896
8HHG	FtsQL	S2	1	99	1.03	1.06	342	0.529	0.707	0.964	1.120	0.90	1.240	1.000
8HHH	FtsQL	S2	3	74	0.786	0.826	18.9	0.782	0.459	0.757	0.740	0.79	0.933	3.330
8HHH	FtsQL	S2	1	579	0.896	0.873	350	0.625	0.542	0.950	0.471	1.20	0.392	1.030
2VH1	FtsQ	S3	3	23	0.592	0.572	67.6	0.795	0.536	0.688	0.521	0.61	0.849	2.580
8HHG	FtsQB	S3	2	44	0.738	0.748	135	0.763	0.567	0.629	0.347	0.66	0.525	2.080
8HHH	FtsQB	S3	3	104	0.856	0.89	83.7	0.805	0.482	0.829	0.334	1.04	0.321	1.860
8HHH	FtsQB	S3	1	226	0.895	0.95	233	0.733	0.495	0.808	0.944	0.88	1.070	1.640
8HHG	FtsQBL	S3	2	43	0.733	0.74	138	0.778	0.568	0.626	0.327	0.67	0.490	1.960
8HHH	FtsQBL	S3	1	49	0.737	0.714	187	0.777	0.605	0.704	0.208	0.94	0.221	0.979
6H9N	FtsQ	S4	2	26	0.622	0.566	93	0.755	0.623	0.808	0.429	0.88	0.487	0.906
8HHF	FtsQ	S4	3	38	0.665	0.567	125	0.712	0.618	0.804	0.094	1.17	0.080	0.730
8HHG	FtsQ	S4	3	27	0.625	0.529	94.7	0.654	0.664	0.940	0.510	1.10	0.465	0.626
8HHH	FtsQ	S4	2	29	0.621	0.498	88.2	0.681	0.637	0.903	0.200	1.20	0.167	0.455
8HHF	FtsQB	S4	5	36	0.674	0.503	114	0.700	0.649	0.891	0.041	1.38	0.0294	0.560

8HHG	FtsQB	S4	5	28	0.632	0.532	92.6	0.682	0.664	0.922	0.468	1.12	0.419	0.634
8HHH	FtsQB	S4	6	32	0.639	0.499	93	0.692	0.635	0.894	0.183	1.27	0.144	0.371
8HHG	FtsQBL	S4	3	30	0.625	0.556	93	0.691	0.63	0.824	0.389	1.03	0.377	0.604
8HHH	FtsQBL	S4	3	36	0.664	0.561	98.8	0.664	0.634	0.922	0.282	1.18	0.240	0.447
8HHF	FtsQL	S4	3	44	0.696	0.61	121	0.692	0.613	0.787	0.052	1.17	0.045	0.483
8HHH	FtsQL	S4	4	36	0.664	0.563	99.5	0.670	0.634	0.913	0.278	1.17	0.238	0.439
2VH1	FtsQ	S5	2	30	0.716	0.71	72.7	0.589	0.631	0.871	1.810	0.56	3.230	0.301
8HHH	FtsQ	S5	1	35	0.691	0.606	97.1	0.533	0.684	0.929	0.331	1.11	0.299	3.380
6H9O	FtsQ	S5	1	110	1.02	1.05	239	0.536	0.707	0.965	0.885	0.97	0.910	0.647
5Z2W	FtsQ	S5	2	42	0.822	0.803	88.2	0.500	0.737	1.020	2.360	0.76	3.090	0.544
8HHF	FtsQ	S5	1	70	0.874	0.869	185	0.586	0.673	0.935	0.537	0.99	0.545	2.14
8HHG	FtsQ	S5	1	56	0.826	0.82	153	0.634	0.66	0.845	0.764	0.86	0.893	2.19
8HHH	FtsQB	S5	2	34	0.693	0.606	98.8	0.564	0.696	0.955	0.354	1.10	0.321	2.97
8HHF	FtsQB	S5	1	68	0.897	0.877	155	0.429	0.735	0.997	0.655	1.04	0.629	6.82
8HHG	FtsQB	S5	1	46	0.865	0.839	131	0.452	0.788	1.090	1.260	0.83	1.520	4.70
8HHH	FtsQBL	S5	2	37	0.715	0.62	103	0.486	0.702	0.934	0.224	1.14	0.196	2.30
8HHF	FtsQBL	S5	1	72	0.915	0.906	152	0.410	0.726	1.010	0.715	1.01	0.711	5.70
8HHG	FtsQBL	S5	1	55	0.874	0.844	145	0.481	0.773	1.050	1.270	0.97	1.300	3.02
8HHH	FtsQL	S5	2	35	0.7	0.602	100	0.533	0.698	0.901	0.284	1.14	0.248	2.45
8HHF	FtsQL	S5	2	74	0.934	0.923	156	0.373	0.748	1.030	0.795	1.03	0.774	5.13
8HHG	FtsQL	S5	2	56	0.896	0.883	146	0.456	0.766	1.050	1.270	0.86	1.480	1.91
8HHH	FtsQ	S6	3	21	0.617	0.595	38.8	0.691	0.585	0.918	1.310	0.57	2.270	1.98
8HHF	FtsQ	S6	2	70	0.865	0.911	164	0.738	0.565	0.736	0.628	0.66	0.957	0.911
8HHF	FtsQB	S6	4	31	0.637	0.622	65.5	0.754	0.541	0.729	0.705	0.69	1.020	1.54
8HHG	FtsQB	S6	7	26	0.618	0.586	62.4	0.752	0.576	0.838	0.828	0.73	1.130	0.617
8HHH	FtsQB	S6	5	29	0.645	0.643	71	0.816	0.537	0.641	0.725	0.57	1.270	0.849
8HHG	FtsQB	S6	6	25	0.562	0.365	62.8	0.643	0.591	0.825	0.017	1.41	0.012	0.889
8HHF	FtsQBL	S6	6	22	0.628	0.607	64.8	0.744	0.589	0.86	0.967	0.58	1.680	1.82
8HHH	FtsQBL	S6	5	21	0.615	0.589	46	0.700	0.589	0.903	0.967	0.60	1.620	2.08
8HHG	FtsQBL	S6	7	23	0.557	0.349	61.1	0.667	0.606	0.889	0.024	1.43	0.017	0.849
8HHH	FtsQL	S6	5	21	0.605	0.58	49.7	0.731	0.572	0.836	0.908	0.59	1.530	1.68

8HHG	FtsQL	S6	5	21	0.54	0.334	59.7	0.708	0.604	0.79	0.078	1.41	0.055	0.95
8HHG	FtsQL	S6	3	47	0.746	0.77	91.6	0.777	0.537	0.686	0.807	0.6	1.340	0.64
5Z2W	FtsQ	S8	1	48	0.825	0.727	83.3	0.429	0.773	1.170	0.708	1.19	0.594	0.383
2VH1	FtsQ	S7	4	23	0.586	0.527	52.5	0.657	0.599	0.883	0.744	0.87	0.857	0.674
5Z2W	FtsQ	S7	3	31	0.671	0.695	79.2	0.791	0.512	0.600	0.760	0.41	1.830	1.06
6H9N	FtsQ	S7	1	37	0.643	0.614	75.1	0.702	0.545	0.720	0.454	0.87	0.519	0.471
8HHG	FtsQ	S7	2	28	0.596	0.57	76.1	0.783	0.525	0.688	0.568	0.74	0.764	1.07
8HHG	FtsQB	S7	4	25	0.594	0.563	78.9	0.781	0.547	0.742	0.647	0.72	0.895	0.908
8HHG	FtsQBL	S7	6	25	0.595	0.566	77.9	0.775	0.546	0.733	0.584	0.71	0.824	0.69
8HHG	FtsQL	S7	4	29	0.609	0.595	76.5	0.779	0.512	0.653	0.547	0.67	0.818	0.88
6H9O	FtsQ	S8	2	29	0.643	0.609	80.9	0.642	0.593	0.835	0.709	0.78	0.915	0.95
8HHG	FtsQ	S8	4	23	0.528	0.38	58.3	0.733	0.563	0.749	0.021	1.25	0.017	1.06
8HHF	FtsQB	S8	3	29	0.649	0.615	62.1	0.586	0.602	0.942	1.17	0.77	1.510	1.58
8HHF	FtsQBL	S8	3	30	0.657	0.601	61.7	0.565	0.65	0.997	1.06	0.93	1.140	1.01
8HHF	FtsQL	S8	5	25	0.664	0.615	65.2	0.597	0.673	1.090	0.919	0.79	1.160	1.43
5Z2W	FtsQ	S8	1	48	0.825	0.727	83.3	0.429	0.773	1.170	0.708	1.19	0.594	0.383

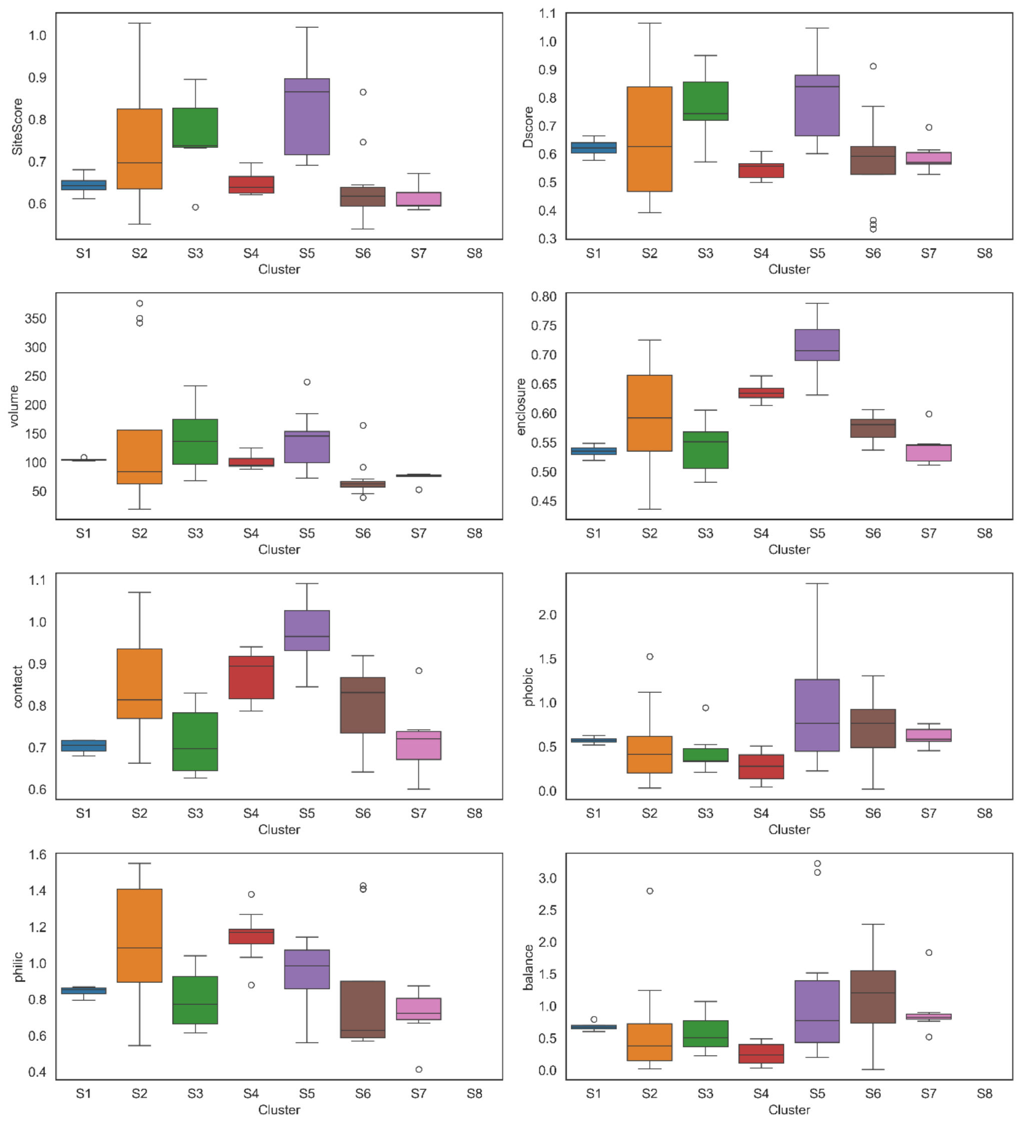

Figure S8. Distribution of features identifed by SiteMap across all eight clusters.

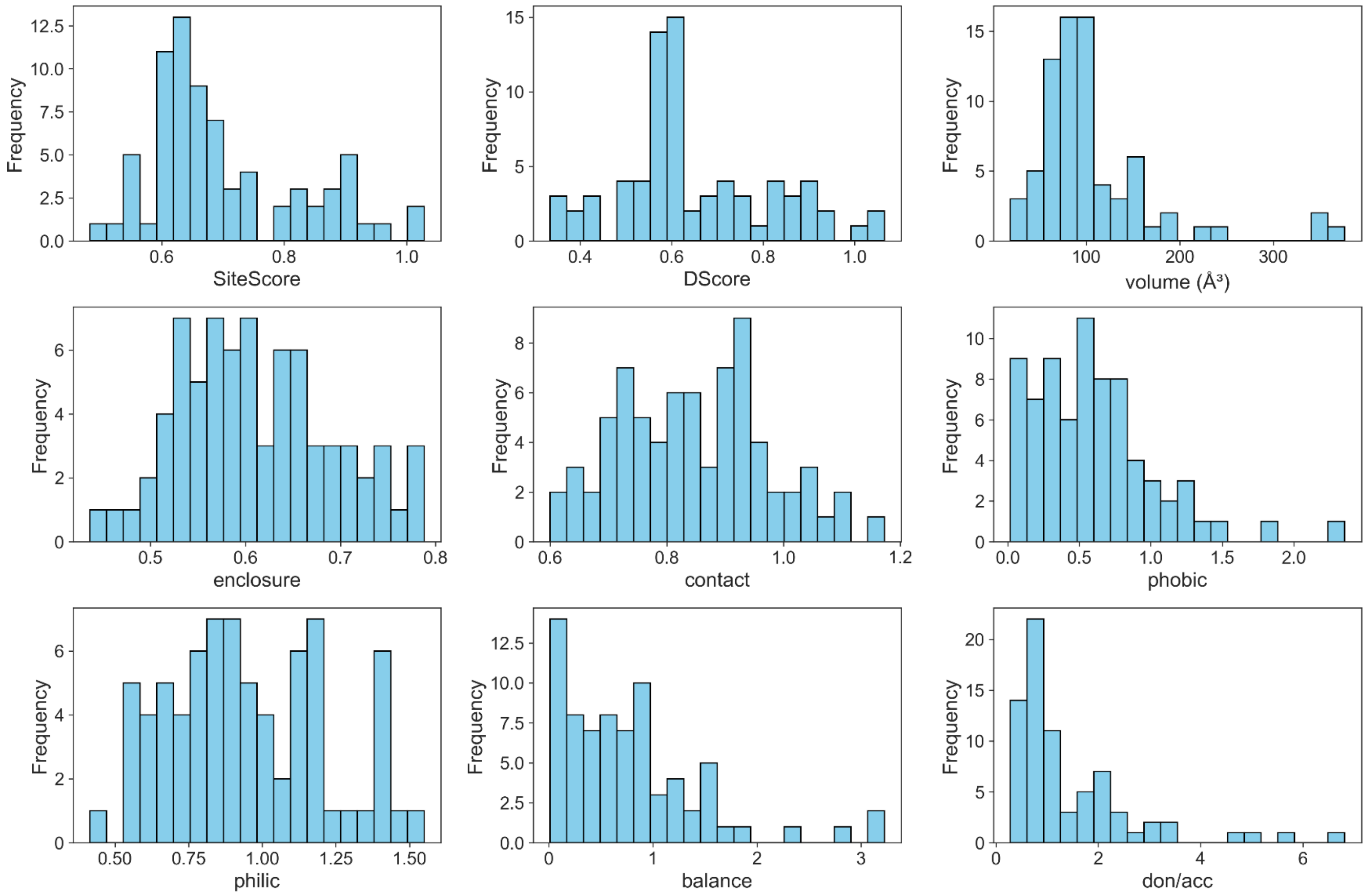

Figure S9. Distribution of features calculated by SiteMap between individual binding sites.

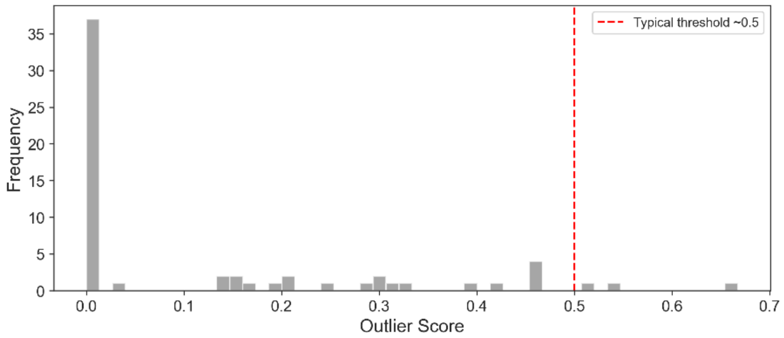

Figure S10. Distribution of HDBSCAN clustering scores.

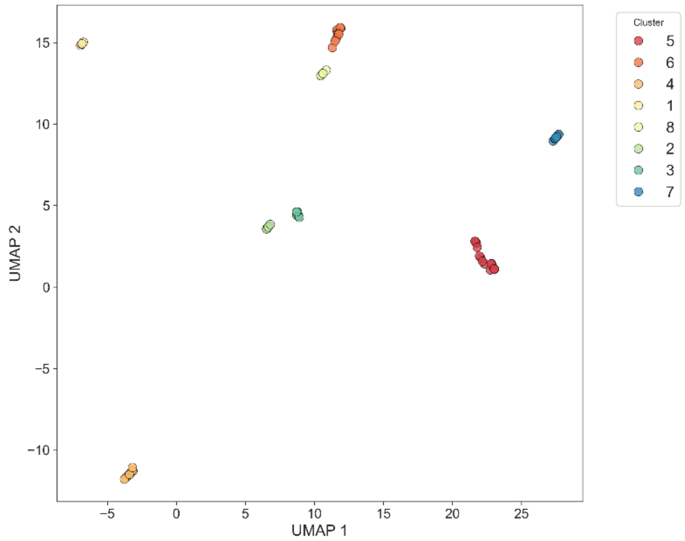

.

Figure S11. 2D UMAP projection (HDBSCAN clusters).

Table S7. Dscore values for all sites across clusters S1-S8.

PDB ID	Complex	S1	S2	S3	S4	S5	S6	S7	S8
2VH1	FtsQ	NA	0.716	0.572	NA	0.71	NA	0.527	NA
5Z2W	FtsQ	NA	NA	NA	NA	0.803	NA	0.695	0.727
6H9N	FtsQ	NA	NA	NA	0.566	NA	NA	0.614	NA
6H9O	FtsQ	NA	NA	NA	NA	1.05	NA	NA	0.609
8HHF	FtsQ	0.578	NA	NA	0.567	0.869	0.911	NA	NA
8HHF	FtsQB	0.612	0.414	NA	0.503	0.877	0.622	NA	0.615
8HHF	FtsQBL	0.664	0.418	NA	NA	0.906	0.607	NA	0.601
8HHF	FtsQL	0.632	1.01	NA	0.61	0.923	NA	NA	0.615
8HHG	FtsQ	NA	NA	NA	0.529	0.82	NA	0.57	0.38
8HHG	FtsQB	NA	0.545	0.748	0.532	0.839	0.586. 0.365	0.563	NA
8HHG	FtsQBL	NA	0.583	0.74	0.556	0.844	0.349	0.566	NA
8HHG	FtsQL	NA	1.06	NA	NA	0.883	0.77. 0.334	0.595	NA
8HHH	FtsQ	NA	NA	NA	0.498	0.606	0.595	NA	NA
8HHH	FtsQB	NA	0.669. 0.392	0.89. 0.95	0.499	0.606	0.643	NA	NA
8HHH	FtsQBL	NA	0.482	0.714	0.561	0.62	0.589	NA	NA
8HHH	FtsQL	NA	0.873	NA	0.563	0.602	0.58	NA	NA

7. Comparisson FTMap vs Sitemap

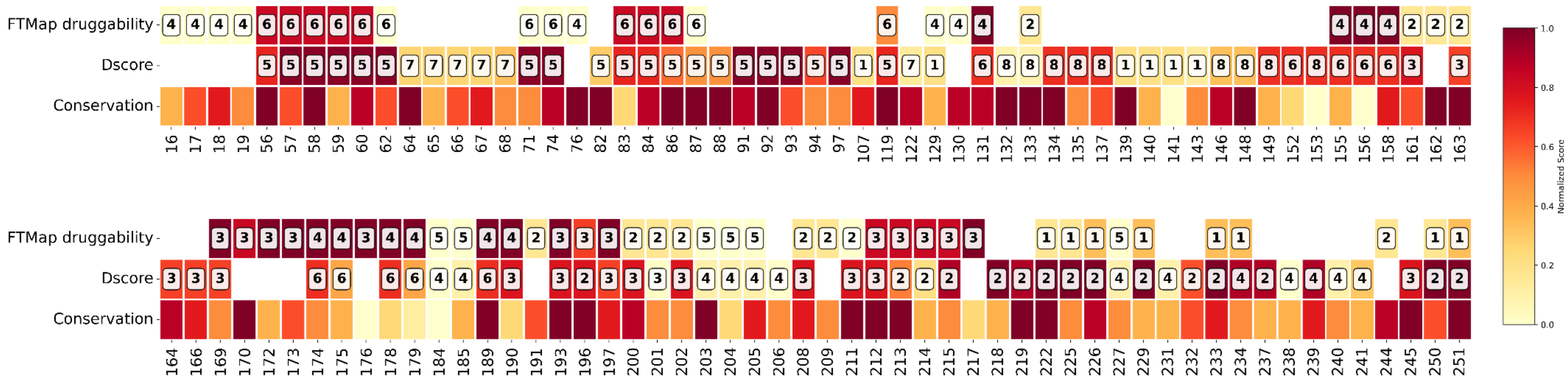

Figure S12. Druggability and evolutionary conservation of amino acid residues identified by FTMap, SiteMap and ConSurf presented as a heatmap. For each residue a normalized score is shown based on FTMap classification. SiteMap DScore or the normalized ConSurf score. Numbers in each cell indicate the corresponding FTMap binding sites (BS1–BS6) and SiteMap sites (S1–S8).

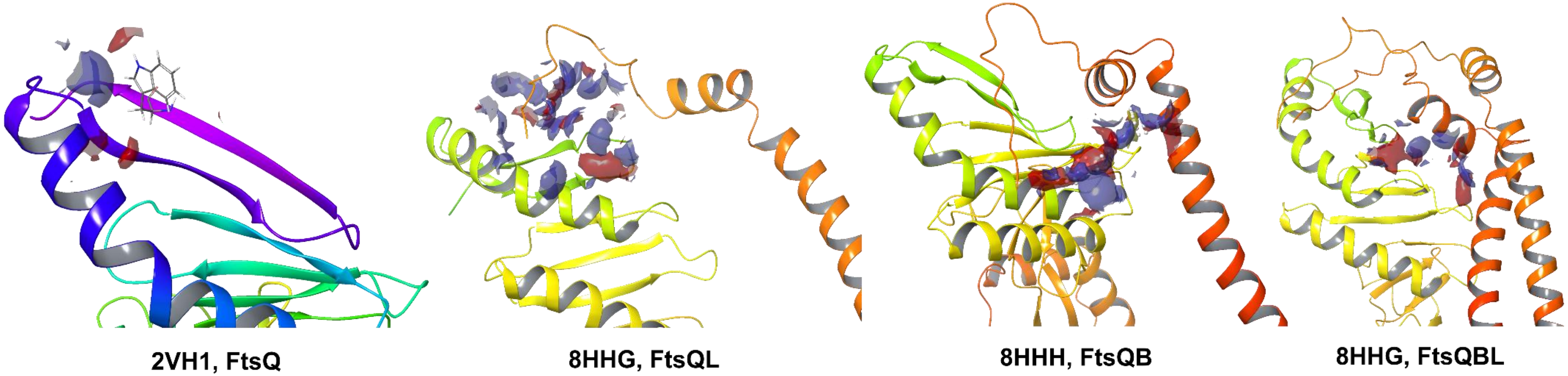

Figure S13. Comparisson of binding sites identified in different complex forms of FtsQBL complex by Sitemap. Each label under structure represents PDB ID and complex.

8. Literature

1. Van Den Ent, F.; Vinkenvleugel, T.M.F.; Ind, A.; West, P.; Veprintsev, D.; Nanninga, N.; Den Blaauwen, T.; Löwe, J. Structural and Mutational Analysis of the Cell Division Protein FtsQ. *Molecular Microbiology* **2008**, *68*, 110–123, doi:10.1111/j.1365-2958.2008.06141.x.
2. Danguole Kureisaite-Ciziene; Kureisaite-Ciziene, D.; Aravindan Varadajan; Varadajan, A.; A. Varadajan; Stephen H. McLaughlin; McLaughlin, S.H.; Marjolein Glas; Glas, M.; Alejandro Montón Silva; et al. Structural Analysis of the Interaction between the Bacterial Cell Division Proteins FtsQ and FtsB. *Mbio* **2018**, *9*, 1–17, doi:10.1128/mbio.01346-18.
3. Choi, Y.; Kim, J.; Yoon, H.-J.; Jin, K.S.; Ryu, S.; Lee, H.H. Structural Insights into the FtsQ/FtsB/FtsL Complex, a Key Component of the Divisome. *Sci Rep* **2018**, *8*, 18061, doi:10.1038/s41598-018-36001-2.
4. Hong Thuy Vy Nguyen; Xiaorui Chen; Claudia Parada; An-Chi Luo; Orion Shih; U-Ser Jeng; Chia-Ying Huang; Yu-Ling Shih; Che Ma Structure of the Heterotrimeric Membrane Protein Complex FtsB-FtsL-FtsQ of the Bacterial Divisome. *Nature communications* **2023**, *14*, doi:10.1038/s41467-023-37543-4.
5. Käshammer, L.; van den Ent, F.; Jeffery, M.; Jean, N.L.; Hale, V.L.; Löwe, J. Cryo-EM Structure of the Bacterial Divisome Core Complex and Antibiotic Target FtsWIQBL. *Nat Microbiol* **2023**, *8*, 1149–1159, doi:10.1038/s41564-023-01368-0.
6. Yang, L.; Chen, Y.; Chang, S.; Shen, C.; Wang, X.; Zhang, C.; Zhang, Z.; Ding, B.-S.; Su, Z.; Dong, H.; et al. Structural Insights into the Activation of the Divisome Complex FtsWIQLB. *Cell Discov* **2024**, *10*, 1–4, doi:10.1038/s41421-023-00629-w.
